# Real-World Cost-Effectiveness of First-Line Gemcitabine Plus Nab-Paclitaxel vs FOLFIRINOX in Patients With Advanced Pancreatic Cancer

**DOI:** 10.1093/jncics/pkac047

**Published:** 2022-06-27

**Authors:** Vanessa Arciero, Jin Luo, Ambica Parmar, Wei Fang Dai, Jaclyn M Beca, Michael J Raphael, Wanrudee Isaranuwatchai, Steven Habbous, Mina Tadrous, Craig C Earle, Jim J Biagi, Nicole Mittmann, Jessica Arias, Scott Gavura, Kelvin K W Chan

**Affiliations:** Department of Medicine, Division of Medical Oncology, Sunnybrook Health Sciences Centre, University of Toronto, Toronto, ON, Canada; Temerty Faculty of Medicine, University of Toronto, Toronto, ON, Canada; ICES, Toronto, ON, Canada; Department of Medicine, Division of Medical Oncology, Sunnybrook Health Sciences Centre, University of Toronto, Toronto, ON, Canada; Temerty Faculty of Medicine, University of Toronto, Toronto, ON, Canada; Temerty Faculty of Medicine, University of Toronto, Toronto, ON, Canada; Canadian Centre for Applied Research in Cancer Control, Toronto, ON, Canada; Canadian Centre for Applied Research in Cancer Control, Toronto, ON, Canada; Ontario Health, Cancer Care Ontario, Toronto, ON, Canada; Department of Medicine, Division of Medical Oncology, Sunnybrook Health Sciences Centre, University of Toronto, Toronto, ON, Canada; Temerty Faculty of Medicine, University of Toronto, Toronto, ON, Canada; Canadian Centre for Applied Research in Cancer Control, Toronto, ON, Canada; St. Michael’s Hospital, University of Toronto, Toronto, ON, Canada; Ontario Health, Cancer Care Ontario, Toronto, ON, Canada; Women’s College Hospital, Toronto, ON, Canada; Department of Medicine, Division of Medical Oncology, Sunnybrook Health Sciences Centre, University of Toronto, Toronto, ON, Canada; Temerty Faculty of Medicine, University of Toronto, Toronto, ON, Canada; Canadian Partnership Against Cancer, Toronto, ON, Canada; Department of Oncology, Queen’s University, Kingston, ON, Canada; Canadian Agency for Drugs and Technologies in Health, Toronto, ON, Canada; Ontario Health, Cancer Care Ontario, Toronto, ON, Canada; Ontario Health, Cancer Care Ontario, Toronto, ON, Canada; Department of Medicine, Division of Medical Oncology, Sunnybrook Health Sciences Centre, University of Toronto, Toronto, ON, Canada; Temerty Faculty of Medicine, University of Toronto, Toronto, ON, Canada; Canadian Centre for Applied Research in Cancer Control, Toronto, ON, Canada; Ontario Health, Cancer Care Ontario, Toronto, ON, Canada

## Abstract

**Background:**

There are no randomized control trials (RCTs) comparing gemcitabine and nab-paclitaxel (Gem-Nab) and fluorouracil, folinic acid, irinotecan, oxaliplatin (FOLFIRINOX) for advanced pancreatic cancer (APC). Although it is well known that RCT-based efficacy often does not translate to real-world effectiveness, there is limited literature investigating comparative cost-effectiveness of Gem-Nab vs FOLFIRINOX for APC. We aimed to examine the real-world cost-effectiveness of Gem-Nab vs FOLFIRINOX for APC in Ontario, Canada.

**Methods:**

This study compared patients treated with first-line Gem-Nab or FOLFIRINOX for APC in Ontario from April 2015 to March 2019. Patients were linked to administrative databases. Using propensity scores and a stabilizing weights method, an inverse probability of treatment weighted cohort was developed. Mean survival and total costs were calculated over a 5-year time horizon, adjusted for censoring, and discounted at 1.5%. Incremental cost-effectiveness ratio and net monetary benefit were computed to estimate cost-effectiveness from the public health-care payer’s perspective. Sensitivity analysis was conducted using the propensity score matching method.

**Results:**

A total of 1988 patients were identified (Gem-Nab: n = 928; FOLFIRINOX: n = 1060). Mean survival was lower for patients in the Gem-Nab than the FOLFIRINOX group (0.98 vs 1.26 life-years; incremental effectiveness = −0.28 life-years [95% confidence interval = −0.47 to −0.13]). Patients in the Gem-Nab group incurred greater mean 5-year total costs (Gem-Nab: $103 884; FOLFIRINOX: $101 518). Key cost contributors include ambulatory cancer care, acute inpatient hospitalization, and systemic therapy drug acquisition. Gem-Nab was dominated by FOLFIRINOX, as it was less effective and more costly. Results from the sensitivity analysis were similar.

**Conclusions:**

Gem-Nab is likely more costly and less effective than FOLFIRINOX and therefore not considered cost-effective at commonly accepted willingness-to-pay thresholds.

Globally, the cost of cancer care is increasing, largely given the rise in cost of anticancer drugs (1). In 2015, the global cost of anticancer drugs and supportive oncology care increased 11.5% to US$107 billion, with anticancer drugs increasing 14.2% to US$83.7 billion (1). Pancreatic cancer remains one of the costliest cancers to treat (2,3), although prognosis is often poor, with a 5-year survival rate of 8% (4). However, the advent of novel anticancer drugs and combinations for advanced pancreatic cancer (APC) has resulted in improved survival, along with increased costs. Gemcitabine and nab-paclitaxel (Gem-Nab) and fluorouracil, folinic acid, irinotecan, oxaliplatin (FOLFIRINOX) have both independently demonstrated statistically significant improvements in overall survival (OS) compared with gemcitabine alone, despite increased toxicity profiles (5-7).

Gem-Nab was publicly funded as first-line treatment for APC in Ontario, Canada, in April 2015, and FOLFIRINOX in November 2011 (8). Recommendations by the Canadian Agency for Drugs and Technologies in Health (CADTH) pan-Canadian Oncology Drug Review (pCODR) Expert Review Committee for Gem-Nab in 2014 were “conditional on the cost-effectiveness being improved to an acceptable level,” as confidential cost-effectiveness estimates were presumed to be less optimistic than those submitted by the manufacturer (9). CADTH noted that given the lack of robust data for direct comparison, naïve indirect comparisons of randomized control trial (RCT) data with “substantial heterogeneity between the two trails, made the results highly unreliable and uncertain” (9). The UK’s National Institute for Health and Care Excellence (NICE) evaluated Gem-Nab in 2017 with a mixed treatment comparison using a fixed effect model (10). NICE suggested that Gem-Nab is more effective and cost-effective than gemcitabine, given an incremental cost-effectiveness ratio (ICER) of £41 000-£46 000 ($70 516-$79 116 2020 CAD) per quality-adjusted life year (QALY) (10). However, when compared with FOLFIRINOX, NICE suggested Gem-Nab is likely to be less effective and not cost-effective (Gem-Nab is dominated by FOLFIRINOX) (10).

There is no direct RCT comparing Gem-Nab with FOLFIRINOX for APC. A recent real-world analysis of APC in Canada determined a median OS of 6.1 vs 9.6 months for patients treated with Gem-Nab and FOLFIRINOX, respectively (OS hazard ratio [HR] = 0.77, 95% confidence interval [CI] = 0.7 to 0.85) (11). This analysis reported a lower median OS for patients treated with Gem-Nab than reported by a pivotal RCT (6.1 vs 8.7 months) (5,6). However, the FOLFIRINOX group experienced more frequent febrile neutropenia-related hospitalizations (Gem-Nab: 3.3%; FOLFIRINOX: 5.8%; *P* = .001) (11).

Drug funding decisions are typically informed by RCTs (12), however, where uncertainty remains because of insufficient evidence or in instances where reevaluation of cost-effectiveness is warranted after initial reimbursement, real-world evidence (RWE) can serve as a valuable source of information (13). To our knowledge, there are currently no available cost-effectiveness analyses that use real-world population-based comparative data between Gem-Nab and FOLFIRINOX. Thus, this study aimed to conduct a real-world cost-effectiveness analysis comparing Gem-Nab with FOLFIRINOX in patients with APC, from the public health-care payer perspective, in Ontario, Canada.

## Methods

### Study Design

Using routinely collected health-care data from Ontario, Canada (population 14 million), a population-based retrospective cohort study was conducted. Research ethics approval was obtained from Sunnybrook Health Sciences Centre research ethics board (Toronto, Ontario).

Data collection and analysis was conducted by ICES (formerly known as the Institute for Clinical Evaluative Sciences), an independent, nonprofit research institute funded by an annual grant from the Ontario Ministry of Health and Long-Term Care. As a prescribed entity under Ontario’s privacy legislation, ICES is authorized to collect and use health-care data for the purposes of health system analysis, evaluation, and decision support. Secure access to these data is governed by policies and procedures that are approved by the Information and Privacy Commissioner of Ontario. To mitigate any risk for re-identification, small cell counts (<6 patients) are repressed herein.

### Cohort Definition

Patients aged 18 years and older prescribed first-line gemcitabine, nab-paclitaxel, irinotecan, or oxaliplatin for APC between April 17, 2015, and March 31, 2019, were identified through Cancer Care Ontario’s New Drug Funding Program (NDFP) database. NDFP reimburses hospitals for intravenous cancer drugs administered according to clinical criteria. Patients who received Gem-Nab or FOLFIRINOX were eligible for inclusion. Patients who received treatment at least 60 days before their date of cancer diagnosis (recorded in the registry), died prior to their index date (date of treatment initiation), or were not an Ontario resident at the time of diagnosis were excluded. Patients were also excluded if they were missing an income quintile, rurality status, extent of disease (locally advanced vs metastatic), or had an Eastern Cooperative Oncology Group performance status (ECOG PS) of at least 2 or missing. Included patients were then linked to the Ontario Cancer Registry where diagnosis of APC was confirmed using the site code C-25 from the International Classification of Diseases for Oncology (third edition). Patients were followed until March 31, 2020.

### Baseline Covariates

Patient characteristics were collected from linked administrative databases. Datasets were linked using unique encoded identifiers and analyzed at ICES. Patients were identified across databases using their Ontario Health Insurance Plan number. Demographic characteristics were obtained from the Registered Persons Database. Using postal codes from the Postal Code Conversion File and 2016 Census [Statistics Canada (14)], neighborhood-level income quintile, health region (Local Health Integration Network), and rurality status were obtained. Extent of disease, topography, and ECOG PS were obtained from NDFP enrollment form and Ontario Cancer Registry. Hospital systemic and radiation treatments were collected from the Activity Level Reporting database, and surgical records in Canadian Institutes for Health Information Discharge Abstract Database (CIHI DAD). Charlson-Deyo comorbidity index and adjusted clinical groups (ACG) categories (ACG System Aggregated Diagnosis Groups, The Johns Hopkins ACG System version 10) were derived from CIHI DAD, CIHI National Ambulatory Care Reporting System, and CIHI Same Day Surgery database. Death records were obtained from Registered Persons Database.

### Propensity Score Weighting

Baseline covariates included as variables in the propensity score calculation (logistic regression) included age, sex, prior cancer diagnosis, Charlson-Deyo comorbidity index, total ACG score (2-year look-back period, not excluding nonpancreatic malignancies), health region (Local Health Integration Network), income quintile, rurality, time from pancreatic cancer diagnosis, prior pancreatic surgery, prior pancreatic radiation, adjuvant chemotherapy (including gemcitabine, FOLFIRINOX, gemcitabine plus capecitabine, folinic acid plus fluorouracil, fluorouracil), extent of disease (locally advanced vs metastatic), ECOG PS, and index year. Using propensity scores and a stabilizing weights method (15,16), an inverse probability of treatment weighting (IPTW) cohort was developed. Propensity-weighted standardized differences were calculated to evaluate the balance between groups. In keeping with currently accepted statistical practices, standardized differences of no more than 0.1 were considered as evidence of acceptable balance (17).

The use of an IPTW cohort was elected to be used for the primary analysis as it provides insight into the average treatment effect among the cohort (12,18). As well, the IPTW cohort analysis includes a larger sample size than the propensity score matched (PSM) cohort (n = 1988 vs 1100, respectively).

### Treatment Effectiveness

Costing data was retrieved from linked administrative databases (Supplementary Table 1, available online). A bottom-up approach was used to compute patient-level costs, as unit costs were combined with patient-level utilization data (19).

Treatment effectiveness was measured using life years (LYs) and QALYs. Survival was calculated for each patient from index date to death or to final date of follow-up (maximum 5 years from index). In instances where patients were alive, but without 5-year follow-up from index, they were censored at the last date of follow-up. QALYs were calculated by adjusting 5-year survival with published health utility weights; 0.8 for preprogression (on treatment) and 0.73 for postprogression (end of treatment to death or final date of follow-up) to align with existing literature and previously evaluated models (10,20-23). To adjust for administrative censoring, inverse probability censoring weighting was used for treatment effectiveness and cost analyses (30-day interval partitioning) (24). Survival was discounted 1.5% annually (25).

### Costs

Five-year total costs were estimated for each patient from the public health-care payer perspective [via costing macro from ICES (26)]. Given the natural history of APC, a 5-year time horizon largely represents a life-time horizon. Total cost was calculated for each patient from index date until end of follow-up for individual cost components using administrative data, including systemic therapy drug acquisition, acute inpatient hospitalization, physician services, ambulatory cancer care, emergency department visits, hospital outpatient clinic operating costs, outpatient oral drug acquisition, home care services, and other costs. CIHI resource intensity weight method was used to calculate costs associated with hospitalization and surgery (27) (Supplementary Table 1, available online). Resource intensity weight is an indicator calculated annually based on data from British Columbia, Alberta, and Ontario and the Discharge Abstract Database (DAD) to represent the expected consumption of health-care resources by the average patient (28). Costs were adjusted to 2019 CAD (Statistics Canada Consumer Price Index) and rounded to the nearest dollar. Costs were discounted 1.5% annually and adjusted using inverse probability censoring weighting (25).

### Cost-Effectiveness Analysis

ICER and incremental net monetary benefit (INMB) were computed to estimate cost-effectiveness. ICER was calculated as incremental cost divided by the incremental effectiveness. 95% confidence intervals of ICERs were estimated using nonparametric bootstrapping with 1000 samples.

INMB was computed using various willingness-to-pay (WTP) thresholds with consideration of total cost and survival by calculating effectiveness for each patient. Net benefit regression was conducted using a linear regression model for INMB (regressed on the treatment variable of Gem-Nab vs FOLFIRINOX) at WTP thresholds beginning at $50 000 per LY and QALY to obtain point estimates and 95% confidence intervals for INMB. A positive INMB suggests that Gem-Nab is more cost-effective than FOLFIRINOX at the respective WTP threshold.

### Sensitivity Analysis

Sensitivity analysis was conducted using a PSM cohort. Propensity scores were used to match groups 1:1, with a caliper width of 0.2 standard deviations (29).

## Results

### Study Population and Baseline Characteristics

**Figure 1.**
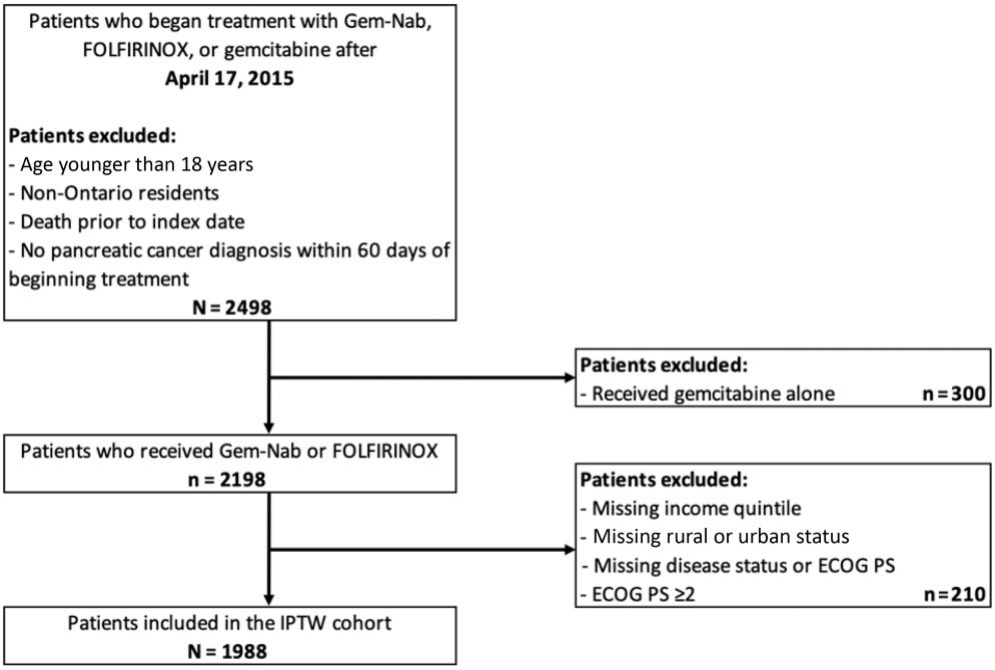
IPTW cohort creation. ECOG PS = Eastern Cooperative Oncology Group Performance Status; FOLFIRINOX = fluorouracil, folinic acid, irinotecan, oxaliplatin; Gem-Nab = gemcitabine plus nab-paclitaxel; IPTW = inverse probability of treatment weighted.

Overall, 2498 patients were originally identified. Patients were excluded for receiving gemcitabine alone (n = 300), missing key demographic data, and/or having an ECOG PS of at least 2 (n = 210). Therefore, 1988 patients (Gem-Nab: n = 928; FOLFIRINOX: n = 1060) were eligible for inclusion in the IPTW cohort (Figure 1).

Mean age of patients was 65.7 and 64.8 years for Gem-Nab and FOLFIRINOX, respectively. In the Gem-Nab group, 42.3% of patients were female and 42.7% within the FOLFIRINOX group. All key characteristics were balanced between groups (Table 1).

**Table 1.** Baseline and IPTW cohort characteristics by treatment

Characteristics	Before IPTW		After IPTW		
	Gemcitabine plus nab-paclitaxel (n = 928)	FOLFIRINOX (n = 1060)	Gemcitabine plus nab-paclitaxel (n = 936.55)	FOLFIRINOX (n = 1046.61)	Weighted standardized difference
Mean age at treatment initiation (SD)	69.2 (9.0)	61.9 (8.8)	65.7 (9.5)	64.8 (9.2)	0.097
Sex, No. (%)					
Female	392 (42.2)	475 (44.8)	395.9 (42.3)	446.7 (42.7)	0.008
Male	536 (57.8)	585 (55.2)	540.6 (57.7)	599.9 (57.3)	0.008	
Tumor site, No. (%)					
Body	141 (15.2)	160 (15.1)	136.2 (14.5)	157.8 (15.1)	0.015
Head	470 (50.6)	564 (53.2)	494.0 (52.7)	560.7 (53.6)	0.017
Tail	147 (15.8)	167 (15.8)	138.7 (14.8)	159.9 (15.3)	0.013
Miscellaneous	170 (18.3)	169 (15.9)	167.8 (17.9)	168.3 (16.1)	0.049
Metastatic disease, vs locally advanced, No. (%)	688 (74.1)	669 (63.1)	643.0 (68.7)	721.0 (68.9)	0.005
Mean days from diagnosis to treatment (SD)	138.4 (300.9)	111.2 (227.9)	134.4 (255.5)	124.3 (299.3)	0.036
Prior pancreatic surgery, No. (%)	142 (15.3)	162 (15.3)	164.6 (17.6)	157.2 (15.0)	0.069
Prior pancreatic radiation, No. (%)	32 (3.4)	34 (3.2)	32.8 (3.5)	35.3 (3.4)	0.007
Prior cancer diagnosis, No. (%)	167 (18.0)	149 (14.1)	145.0 (15.5)	174.0 (16.6)	0.031
ECOG PS 1, vs 0, No. (%)	712 (76.7)	650 (61.3)	645.5 (68.9)	718.3 (68.6)	0.006
Charlson-Deyo comorbidity index, No. (%)					
0	307 (33.1)	376 (35.5)	329.4 (35.2)	367.1 (35.1)	0.002
1	167 (18.0)	179 (16.9)	166.3 (17.8)	183.6 (17.5)	0.006
≥2	81 (8.7)	64 (6.0)	71.5 (7.6)	80.7 (7.7)	0.003
Unknown^a^	373 (40.2)	441 (41.6)	369.3 (39.4)	415.2 (39.7)	0.005
ACG category, No. (%)					
0-4	57 (6.1)	88 (8.3)	59.3 (6.3)	70.8 (6.8)	0.018
5-9	477 (51.4)	594 (56.0)	496.4 (53.0)	559.7 (53.5)	0.009
10-14	339 (36.5)	351 (33.1)	338.3 (36.1)	370.5 (35.4)	0.015
≥15	55 (5.9)	27 (2.5)	42.6 (4.5)	45.7 (4.4)	0.009
Urban, No. (%)	824 (88.8)	932 (87.9)	823.9 (88.0)	916.9 (87.6)	0.011
Income quintile, No. (%)					
1 (lowest)	177 (19.1)	127 (12.0)	138.8 (14.8)	153.8 (14.7)	0.003
2	200 (21.6)	220 (20.8)	181.2 (19.3)	226.8 (21.7)	0.058
3	177 (19.1)	201 (19.0)	189.2 (20.2)	200.4 (19.1)	0.026
4	185 (19.9)	231 (21.8)	210.6 (22.5)	215.4 (20.6)	0.046
5 (highest)	189 (20.4)	281 (26.5)	216.9 (23.2)	250.1 (23.9)	0.018

a No hospitalization in the look-back period to calculate the Charlson-Deyo comorbidity index. ACG = adjusted clinical groups; ECOG PS = Eastern Co-operative Oncology Group performance status; FOLFIRINOX = fluorouracil, folinic acid, irinotecan, oxaliplatin; IPTW = inverse probability treatment weighting.

### Treatment Effectiveness

Mean treatment effectiveness was lower for patients in the Gem-Nab group than the FOLFIRINOX group (0.98 vs 1.26 LYs, respectively). Mean incremental effectiveness between Gem-Nab and FOLFIRINOX was −0.28 (95% CI = −0.47 to −0.13) LYs (Table 2). When accounting for health utilities, mean incremental effectiveness between Gem-Nab and FOLFIRINOX was -0.21 (0.75 vs 0.96) QALY, respectively.

**Table 2.** Costs of treatment, LYG, and QALY in the IPTW cohort^a^

Category	Gemcitabine plus nab-paclitaxel	FOLFIRINOX	Incremental difference^b^
Mean total cost, $ (95% CI)	103 884	101 518	2366 (-8851 to 12 200)
Systemic therapy drug acquisition	13 618	3647	9971
Acute inpatient hospitalization	16 602	18 901	−2300
Physician services	10 974	12 091	−1117
Ambulatory cancer care	40 053	35 956	4098
Emergency department visits	1527	1705	−178
Hospital outpatient clinic operating costs	5131	6275	−1144
Outpatient oral drug acquisition	4939	8476	−3538
Home care services	8549	11 254	−2705
Other	2491	3213	−722
Mean LYG (95% CI)	0.98	1.26	−0.28 (−0.47 to −0.13)
Mean QALY	0.75	0.96	−0.21
Incremental cost-effectiveness ratio ($/LYG)	Gemcitabine plus nab-paclitaxel dominated by FOLFIRINOX		

a All costs have been rounded to the nearest dollar. CI = confidence interval; FOLFIRINOX = fluorouracil, folinic acid, irinotecan, oxaliplatin; IPTW = inverse probability treatment weighting; LYG = life-years gained; QALY = quality adjusted life-years.

b Incremental difference calculated as gemcitabine plus nab-paclitaxel minus FOLFIRINOX.

### Cost Distribution

Gem-Nab was associated with a greater mean total cost than FOLFIRINOX ($103 884 vs $101 518, respectively). Mean incremental cost difference was $2366 (95% CI = −$8851 to $12 200) (Table 2).

### Cost Effectiveness

**Figure 2.**
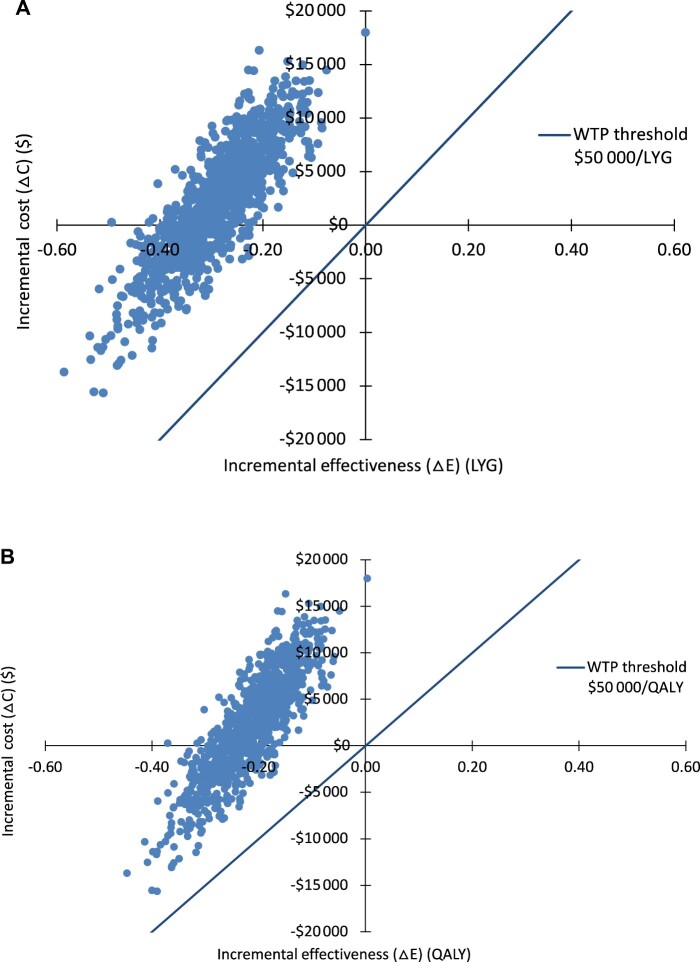
Incremental cost vs effectiveness in the IPTW cohort in **(A)** LYG and **(B)** QALY. IPTW = inverse probability treatment weighting; LYG = life-years gained; QALY = quality adjusted life-years; WTP = willingness to pay.

Gem-Nab was dominated by FOLFIRINOX (Table 2). All bootstrapped samples were in the northwest and southwest quadrants, and 66% of samples had positive incremental cost values (Figure 2). Cost-effectiveness acceptability curve demonstrated that at a WTP threshold of $50 000 per LY, the probability that Gem-Nab is cost-effective is near zero (Supplementary Figure 1, available online).

### Incremental Net Monetary Benefit

**Figure 3.**
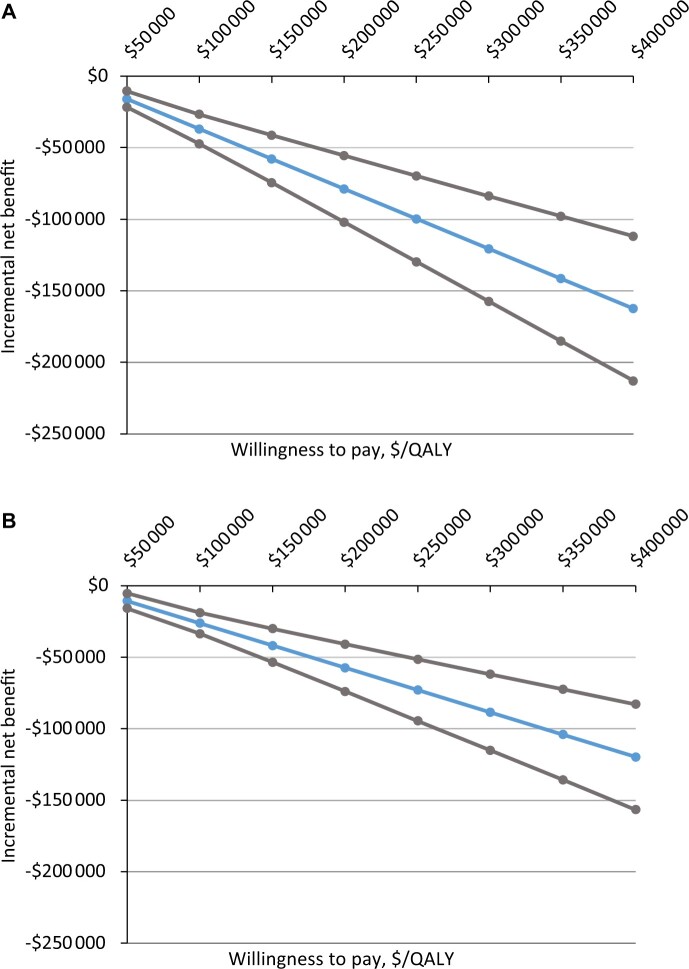
Incremental net monetary benefit in the IPTW cohort for **(A)** LYG and **(B)** QALY. IPTW = inverse probability treatment weighting; LYG = life-years gained; QALY = quality adjusted life-years.

Across all WTP thresholds, the INMB per patient for Gem-Nab (LYs, QALYs) was negative when compared with FOLFIRINOX (Figure 3; Supplementary Table 4, available online).

### Sensitivity Analysis

Following propensity score calculation, 1100 patients (550 per group) were included in the PSM cohort (Supplementary Table 2, available online).

Results from the PSM cohort were similar to the IPTW cohort. Mean 5-year cost of treatment was higher for the Gem-Nab than the FOLFIRINOX group ($103 244 vs $101 446, respectively), and mean effectiveness was less (Gem-Nab = 0.95 LYs, 0.73 QALYs; FOLFIRINOX = 1.20 LYs, 0.92 QALYs). Therefore, Gem-Nab was dominated by FOLFIRINOX. Across all WTP thresholds, the INMB per patient was negative for Gem-Nab when compared with FOLFIRINOX (Supplementary Figures 2-4, Supplementary Tables 3 and 4, available online).

## Discussion

In this real-world cost-effectiveness analysis comparing Gem-Nab with FOLFIRINOX, 1988 patients diagnosed with APC in Ontario, Canada, were analyzed using an IPTW cohort. Mean treatment effectiveness was lower for patients in the Gem-Nab than the FOLFIRINOX group (0.98 LYs, 0.75 QALYs vs 1.26 LYs, 0.96 QALYs, respectively). Gem-Nab was also associated with a higher mean total cost ($103 884 vs $101 518, respectively). Gem-Nab was dominated by FOLFIRINOX in the base case and sensitivity analysis.

A recent Canadian cost-effectiveness analysis (20) conducted through a 3-state Markov model (using data from a RCT-based network meta-analysis) (30) yielded an incremental cost of $54 043 CAD and 0.814 life-years gained (LYG) ($66 391/LYG) for Gem-Nab and $26 443 CAD and 1.006 LYG ($26 285/LYG) for FOLFIRINOX, both vs gemcitabine, suggesting increased costs and lower survival for Gem-Nab. Other jurisdictions have conducted similar models, such as Cui et al. (21) and Zhou et al. (31), who independently evaluated Gem-Nab vs FOLFIRINOX from a Chinese perspective. Although both suggested Gem-Nab was associated with lower costs than FOLFIRINOX, they reported conflicting survival benefits [Gem-Nab = 1.78 LY; FOLFIRINOX = 1.07 LY (21); Gem-Nab = 0.51 QALY; FOLFIRINOX = 0.67 QALY (31)]. Nonetheless, these models were based on estimations of resource utilization and costs from published literature, which has inherent limitations, such as structural uncertainty of the model based on assumptions, or challenges associated with choices of input parameters from multiple, potentially noncomparable, sources of patient cohorts that may not be generalizable to the routine patient population.

Existing literature offers a wide range of results when attempting to quantify the cost-effectiveness of Gem-Nab vs FOLFIRINOX. Economic review by CADTH pCODR and NICE both cite uncertainty in evaluation given the lack of robust direct comparisons (9,10), however, both suggest that Gem-Nab is likely to be dominated by FOLFIRINOX. Nonetheless, other independent model-based analyses yield differing results (20,21,31-33). There are several factors that may contribute to contrasting results from previous model-based analyses, including uncertainty in model, differing data sources, and various drug and related health-care costs.

In Canada, the period of data protection for oxaliplatin (component of FOLFIRINOX) ended in 2015 (34), allowing for the introduction of generic brand competitors, the first of which was funded in Ontario in February 2016 (market date December 2015). Over time, numerous oxaliplatin generics have been funded, creating opportunities for cost savings via increased competition and reduced investment from manufacturers in discovery and clinical trials (35).

To our knowledge, this analysis is the first to use RWE to evaluate the cost-effectiveness of Gem-Nab vs FOLFIRINOX. The use of RWE is an inherent strength, as our results are population based, use individual patient data, and include all patients who are clinically eligible for treatment with Gem-Nab and FOLFIRINOX. Our analysis includes a broad range of cost components, potentially beyond what would be included in model-based analyses, including costs associated with management of serious adverse events, without needing assumptions. Additionally, this analysis applied robust statistical techniques to adjust for differences between groups and address censoring. Typically, drug funding decisions are based on clinical benefit reported in RCTs and model-based economic estimations of drug value (12). However, this may result in uncertainty in assessment, as RCT data itself of generalizability can be limited to the average cancer patient (13), contributing to the discordance between RCT-based efficacy and real-world effectiveness (36-43). RWE is valuable in not only offering an evaluation of real-world drug effectiveness but, when used for cost-assessment, may allow for opportunities to revise existing funding mechanisms postmarket. RWE can be used to reassess previous drug funding recommendations and provide updated guidance to decision makers (44), allowing for drug-price renegotiation and potentially increased funding opportunities for alternatives (13).

Because of the nonrandomized nature of the study design, there is inherent risk of confounding by indication. Using propensity scores to generate IPTW and PSM cohorts, groups were balanced on measured characteristics. Nonetheless, there may be risk of additional unmeasured variables that may contribute to residual confounding, such as symptom burden (45), surgical margins, biomarkers (serum Ca19-9), radiographic volume of burden of disease, and other clinical factors, as well as educational attainment and other health behaviors. Additionally, costs and survival were calculated over a 5-year time horizon. Generally, 5 years represent a life-time horizon for patients with APC and the time horizon used by pCODR’s Economic Guidance Report (46), but there are rare instances where individuals may survive beyond 5 years. In these circumstances, our measurements may be an underestimation, albeit a small one. Our calculated costs may be greater than those truly incurred by the health-care system, particularly for systemic therapy acquisition costs. This is because reported costs do not consider confidential negotiations, discounts, or rebates that often contribute to overall price reductions, which is an inherent issue for all cost-effectiveness analyses and not unique to our study (12). Changes in cost over time and variation in cost censoring may also impact our calculated costs. Finally, to assess survival in QALYs, adjustments were made using published health utility weights. Despite our use of health utilities that align with existing literature, there is inherent risk that these values may not fully represent real-world health utilities.

Our results suggest that for patients with APC with an ECOG PS of 0-1, Gem-Nab is dominated by FOLFIRINOX. Base-case analysis suggests that Gem-Nab offers a smaller survival benefit and is likely to be slightly more costly than FOLFIRINOX. Existing model-based cost-effectiveness analyses report a wide range of findings; however, our results are in line with the general conclusions drawn by pCODR and NICE. FOLFIRINOX appears to be superior in providing survival benefits at a lower cost, but there is also likely a subset of Gem-Nab patients for whom may not be suitable to receive FOLFIRINOX (such as those with an ECOG PS ≥2). Overall, as the landscape of oncology therapeutics continues to rapidly evolve, treatment and drug funding decisions will remain challenging. Therefore, reevaluation of cost-effectiveness using RWE is valuable in reassessing and revising existing drug funding decisions initially based on RCT and model-derived evidence.

## Funding

This work was supported by the Canadian Institutes of Health Research (Grant #HRC-154126).

## Notes

**Role of the** **funder:** The funder had no role in design of the study; the collection, analysis, and interpretation of the data; the writing of the manuscript; or the decision to submit the manuscript for publication.

**Disclosures:** No authors have actual or perceived conflicts of interest to disclose.

**Author** **contributions:** Conceptualization: JMB, WI, KWWC; Data Curation: JL; Formal Analysis: JL; Funding Acquisition: KKWC; Investigation: All named authors; Methodology: WI, JMB, JL, WFD, KKWC; Project Administration: VA, JL, KKWC; Resources: JL, KKWC; Supervision: KKWC; Validation: JL; Visualization: VA, JL; Writing—Original Draft: VA, KKWC; Writing—Review & Editing: All named authors.

**Acknowledgements:** ICES is funded by an annual grant from the Ontario Ministry of Health and the Ministry of Long-Term Care. The Canadian Centre for Applied Research in Cancer Control (ARCC) is funded by the Canadian Cancer Society Grant #2020-706936. Parts of this material are based on data and information compiled and provided by Cancer Care Ontario, Ontario’s Ministry of Health and Long-Term Care, and Canadian Institutes for Health Information. Parts of this material are based on data and information provided by Ontario Health (OH). The opinions, results, view, and conclusions reported in this paper are those of the authors and do not necessarily reflect those of OH. No endorsement by OH is intended or should be inferred. Postal Code Conversion File (PCCF), Reference Guide, 2016 Census and Statistics Canada 2019 Consumer Price Index data were adapted from Statistics Canada. This does not constitute an endorsement by Statistics Canada of this product. We thank IQVIA Solutions Canada Inc for use of their Drug Information File. Authors would like to acknowledge the contributions of Erind Dvorani.

**Disclaimers:** The analyses, conclusions, opinions, and statements expressed herein are solely those of the authors and do not reflect those of the funding or data sources; no endorsement is intended or should be inferred.

**Prior presentations:** This work was previously presented, in part, at the 2022 American Society of Clinical Oncology (ASCO) Gastrointestinal Cancers Symposium and 2022 Canadian Centre for Applied Research in Cancer Control Conference.

## Supplementary Material

pkac047_Supplementary_Data

## Data Availability

The dataset from this study is held securely in coded form at ICES. While legal data sharing agreements between ICES and data providers (eg, health-care organizations and government) prohibit ICES from making the dataset publicly available, access may be granted to those who meet prespecified criteria for confidential access, available at www.ices.on.ca/DAS (email: das@ices.on.ca). The full dataset creation plan and underlying analytic code are available from the authors upon request, understanding that the computer programs may rely on coding templates or macros that are unique to ICES and are therefore either inaccessible or may require modification.
