# Supplementary material for: Real-World Cost-Effectiveness of First-Line Gemcitabine Plus Nab-Paclitaxel vs FOLFIRINOX in Patients With Advanced Pancreatic Cancer
Source: JNCI Cancer Spectr. 2022 Jun 27;6(4):pkac047. doi: 10.1093/jncics/pkac047 (PMC9346632; doi:10.1093/jncics/pkac047)
Supplement: pkac047_Supplementary_Data [file pkac047_supplementary_data.pdf]

SUPPLEMENTARY MATERIALS

Supplementary Table 1. Data sources

Category		Data Source
Patient Identifier		Ontario Health Insurance Plan
Cohort Characteristics		
Patient demographics		Registered Persons Database
Disease characteristics		Ontario Cancer Registry<br>New Drug Funding Program
Treatment history		Activity Level Reporting<br>Canadian Institutes for Health Information Discharge Abstract Database
Survival		
Death Records		Registered Persons Database
Cost Category	Individual Cost Component	
Systemic therapy drug acquisition		New Drug Funding Program
Acute inpatient hospitalization		Canadian Institutes for Health Information Discharge Abstract Database
Physician services	Physician fee for service billings	Ontario Health Insurance Plan
	Emergency department shadow billings	
	Medical oncologist shadow billings	
	Family doctor shadow billings	
	Radiation oncologist shadow billings	
	Other physician shadow billings	
Ambulatory cancer care		Canadian Institutes for Health Information National Ambulatory Care Reporting System
Emergency department visits		Canadian Institutes for Health Information National Ambulatory Care Reporting System
Hospital outpatient clinic operating costs		Ontario Health Insurance Plan
Outpatient oral drug acquisition		Ontario Drug Benefit Database
Home care services		Home Care Database
Other	Same day surgery	Canadian Institutes for Health Information Same Day Surgery Database

	Ambulatory dialysis	Canadian Institutes for Health Information National Ambulatory Care Reporting System
	Rehabilitation	Canadian Institutes for Health Information National Rehabilitation Reporting System
	Complex and continuing care	Complex Continuing Care Reporting System
	Long term care	Complex Continuing Care Reporting System Long-Term Care Database<br>Ontario Health Insurance Plan<br>Ontario Drug Benefit Database
	Laboratory billings	Ontario Health Insurance Plan
	Non-physician billings	Ontario Health Insurance Plan
	Family doctor capitation cost	Client Agency Program Enrolment<br>Ontario Health Insurance Plan
	Mental health admissions	Ontario Mental Health Reporting System
	Assistive devices	Assistive Devices Program Database

Supplementary Table 2. Baseline and PSM cohort characteristics by treatment

Characteristics	Before PSM		After PSM		
	Gemcitabine + nab-paclitaxel (n=928)	FOLFIRINOX (n=1,060)	Gemcitabine + nab-paclitaxel (n=550)	FOLFIRINOX (n=550)	Weighted Standardized Difference
Mean age at treatment initiation (SD)	69.2 (9.0)	61.9 (8.8)	65.4 (8.2)	64.7 (8.1)	0.09
Sex, No. (%)					
Female	392 (42.2)	475 (44.8)	241 (43.8)	235 (42.7)	0.02
Male	536 (57.8)	585 (55.2)	309 (56.2)	315 (57.3)	
Tumour site, No. (%)					
Body	141 (15.2)	160 (15.1)	82 (14.9)	74 (13.5)	0.04
Head	470 (50.6)	564 (53.2)	278 (50.5)	298 (54.2)	0.07
Tail	147 (15.8)	167 (15.8)	88 (16.0)	90 (16.4)	0.01
Miscellaneous	170 (18.3)	169 (15.9)	102 (18.5)	88 (16.0)	0.07
Metastatic disease (vs locally advanced), No. (%)	688 (74.1)	669 (63.1)	393 (71.5)	389 (70.7)	0.02
Mean days from diagnosis to treatment (SD)	138.4 (300.9)	111.2 (227.9)	119.4 (216.4)	124.4 (271.6)	0.02
Prior pancreatic surgery, No. (%)	142 (15.3)	162 (15.3)	82 (14.9)	93 (16.9)	0.05
Prior pancreatic radiation, No. (%)	32 (3.4)	34 (3.2)	14 (2.5)	20 (3.6)	0.06
Prior cancer diagnosis, No. (%)	167 (18.0)	149 (14.1)	77 (14.0)	90 (16.4)	0.07
ECOG PS 1 (vs 0), No. (%)	712 (76.7)	650 (61.3)	391 (71.1)	385 (70.0)	0.02
Charlson-Deyo comorbidity index, No. (%)					
0	307 (33.1)	376 (35.5)	178 (32.4)	193 (35.1)	0.06
1	167 (18.0)	179 (16.9)	101 (18.4)	95 (17.3)	0.03
2+	81 (8.7)	64 (6.0)	40 (7.3)	43 (7.8)	0.02
Unknown[a]	373 (40.2)	441 (41.6)	231 (42.0)	219 (39.8)	0.04
ACG Category, No. (%)					
0-4	57 (6.1)	88 (8.3)	39 (7.1)	39 (7.1)	0
5-9	477 (51.4)	594 (56.0)	299 (54.4)	281 (51.1)	0.07
10-14	339 (36.5)	351 (33.1)	191 (34.7)	217 (39.5)	0.1
15+	55 (5.9)	27 (2.5)	21 (3.8)	13 (2.4)	0.08
Urban, No. (%)	824 (88.8)	932 (87.9)	486 (88.4)	486 (88.4)	0
Income Quintile, No. (%)					
1 (lowest)	177 (19.1)	127 (12.0)	95 (17.3)	92 (16.7)	0.01

2	200 (21.6)	220 (20.8)	107 (19.5)	107 (19.5)	0
3	177 (19.1)	201 (19.0)	114 (20.7)	111 (20.2)	0.01
4	185 (19.9)	231 (21.8)	114 (20.7)	107 (19.5)	0.03
5 (highest)	189 (20.4)	281 (26.5)	120 (21.8)	133 (24.2)	0.06

[a]No hospitalization in the lookback period to calculate Charlson-Deyo comorbidity index
PSM, Propensity score matched; SD, standard deviation; ECOG PS, Eastern Co-operative Oncology group performance status; ACG, adjusted clinical groups

Supplementary Table 3. Costs of treatment, LYG, and QALY in the PSM cohort[a]

Category	Gemcitabine + nab-paclitaxel	FOLFIRINOX	Incremental Difference[b]
Mean total cost ($) (95% CI)	103,244	101,446	1,798 (-7,968, 12,620)
Systemic therapy drug acquisition	13,824	3,496	10,328
Acute inpatient hospitalization	16,163	19,272	-3,110
Physician services	10,970	11,860	-890
Ambulatory cancer care	39,842	36,149	3,693
Emergency department visits	1,525	1,625	-101
Hospital outpatient clinic operating costs	5,040	5,809	-769
Outpatient oral drug acquisition	4,862	8,563	-3,701
Home care services	8,763	11,535	-2,771
Other	2,255	3,137	-882
Mean LYG (95% CI)	0.95	1.20	-0.25 (-0.39, -0.086)
Mean QALY	0.73	0.92	-0.18

[a]All costs have been rounded to the nearest dollar
[b]Incremental difference calculated as gemcitabine + nab-paclitaxel minus FOLFIRINOX
LYG, life-years gained; QALY, quality adjusted life-years; PSM, propensity score matched; CI, Confidence interval

Supplementary Table 4. Incremental net monetary benefit in the IPTW and PSM cohorts[a]

Willingness to Pay Threshold	Per Patient (LYG) ($)		Per Patient (QALY) ($)	
	Point Estimate	95% CI	Point Estimate	95% CI
IPTW Cohort				
$50,000	-15,983	-21648, -10,318	-10,633	-15,881, -5,384
$100,000	-36,917	-47,200, -26,633	-26,216	-33,637, -18,796
$150,000	-57,850	-74,437, -41,263	-41,800	-53,492, -30,108
$200,000	-78,784	-102,027, -55,541	-57,383	-73,890, -40,877
$250,000	-99,718	-129,736, -69,700	-72,967	-94,470, -51,464
$300,000	-120,652	-157,499, -83,805	-88,551	-115,129, -61,972
$350,000	-141,586	-185,289, -97,882	-104,134	-135,829, -72,439
$400,000	-162,520	-213,096, -111,943	-119,718	-156,553, -82,882
PSM Cohort				
$50,000	-14,085	-206,20, -7,550	-10,874	-17,697, -4,051
$100,000	-26,371	-36,892, -15,851	-19,950	-27,544, -12,355
$150,000	-38,658	-56,705, -20,611	-29,025	-41,481, -16,570
$200,000	-50,944	-77,169, -24,720	-38,101	-56,512, -19,691
$250,000	-63,231	-97,826, -28,636	-47,177	-71,857, -22,497
$300,000	-75,517	-118,563, -32,472	-56,253	-87,326, -25,179
$350,000	-87,804	-139,341, -36,267	-65,328	-102,857, -27,800
$400,000	-100,090	-160,143, -40,038	-74,404	-118,422, -30,387

[a]All costs have been rounded to the nearest dollar

CI, Confidence interval; IPTW, inverse probability treatment weighting; PSM, propensity score matched; LYG, life-years gained; QALY, quality adjusted life-years

Supplementary Figure 1. Cost-effectiveness acceptability curve in the IPTW cohort in (A) LYG, (B) QALY

(A)

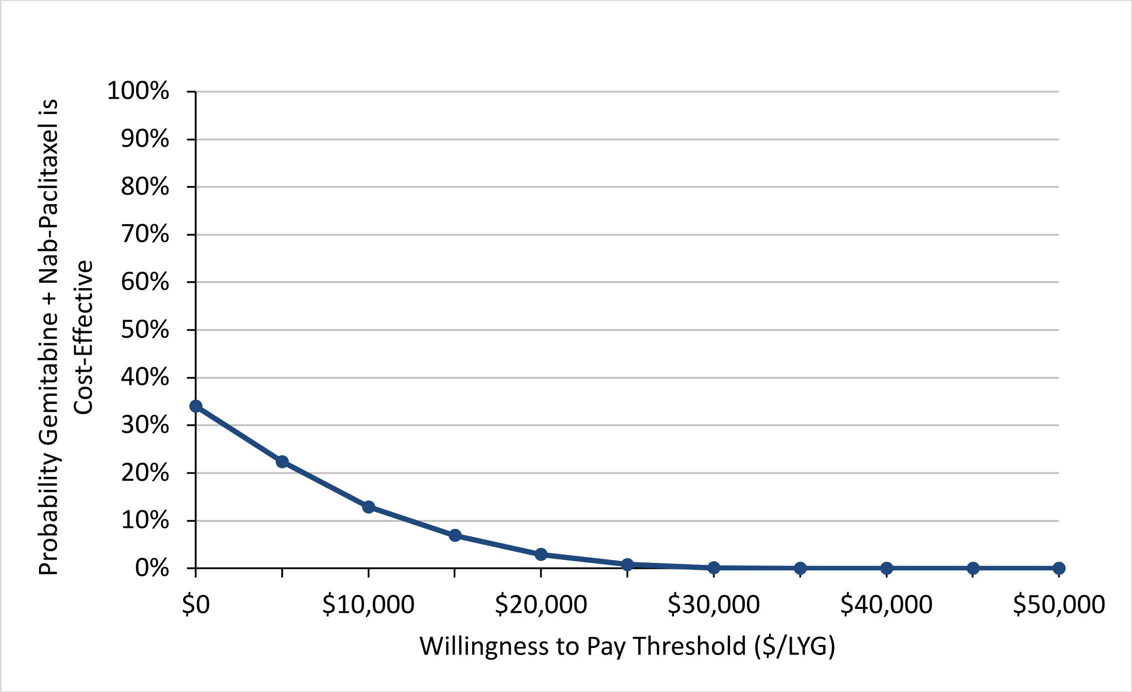

(B)

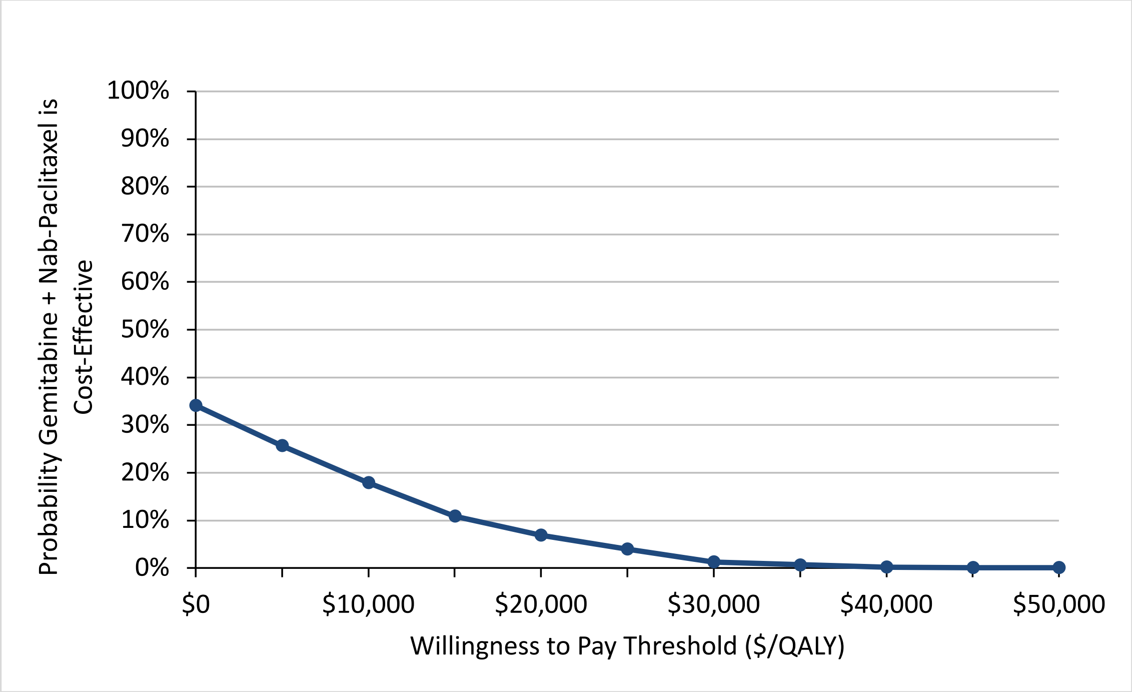

IPTW, Inverse probability treatment weighting; LYG, life-year gained; QALY, quality adjusted life-years

Supplementary Figure 2. Incremental cost versus effectiveness in the PSM cohort in (A) LYG, (B) QALY

(A)

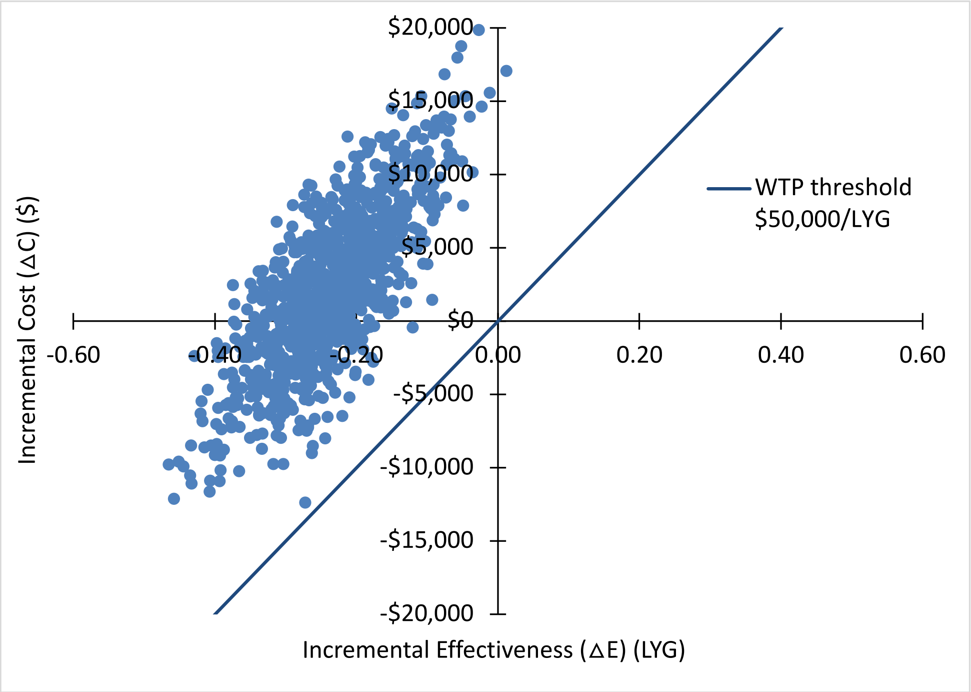

(B)

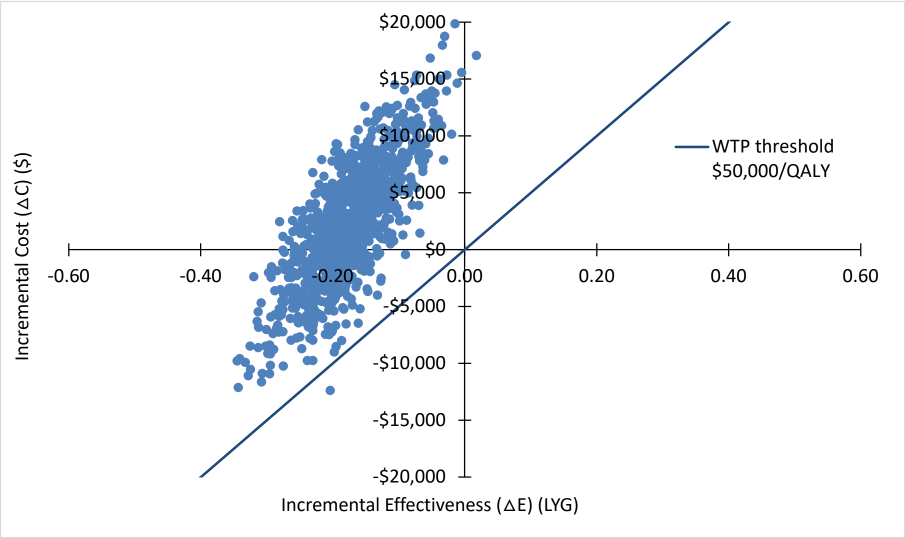

WTP, willingness to pay; LYG, life-years gained; PSM, propensity score matched; QALY, quality adjusted life-years

Supplementary Figure 3. Cost-effectiveness acceptability curve in the PSM cohort in (A) LYG, (B) QALY

(A)

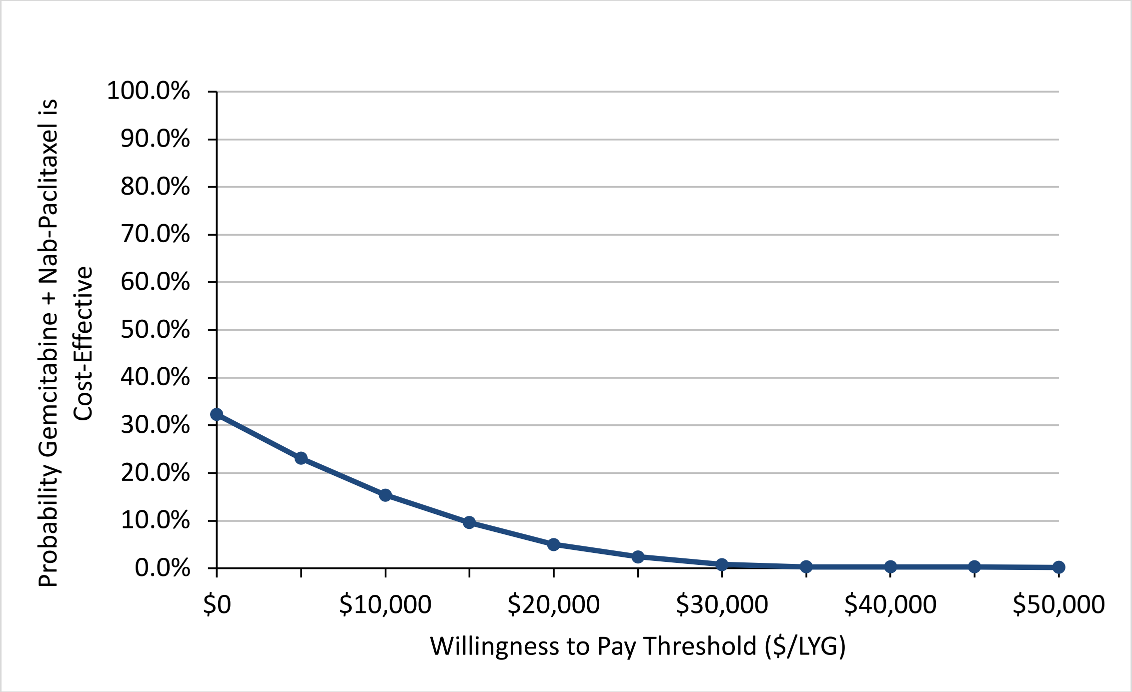

(B)

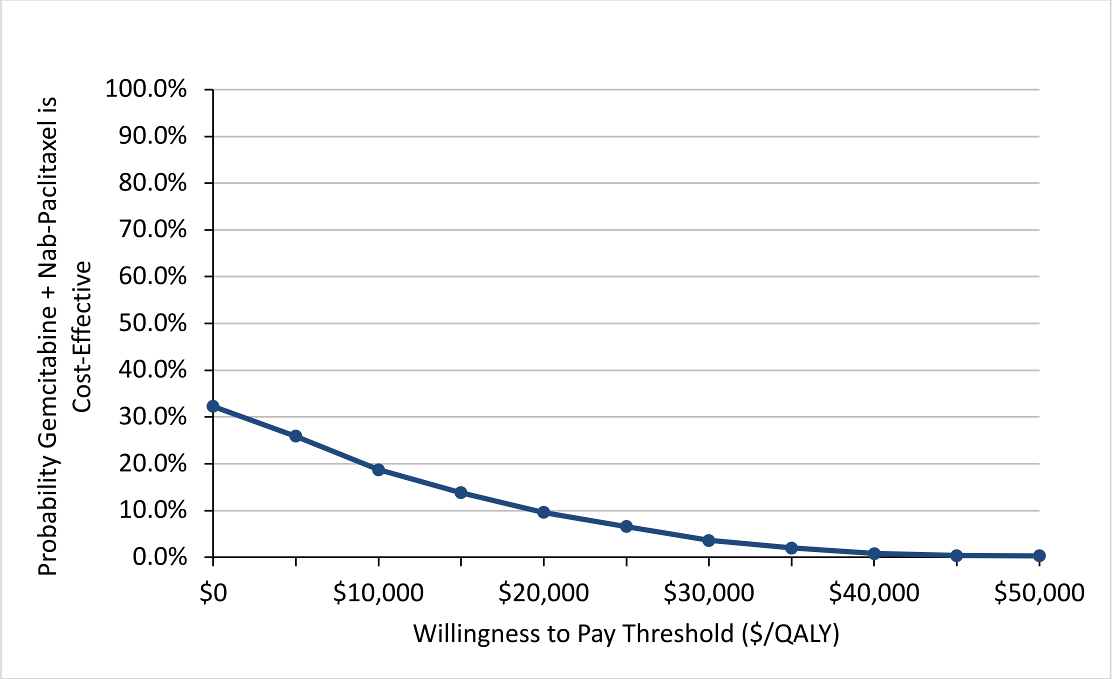

PSM, Propensity score matched; LYG, life-years gained; QALY, quality adjusted life-years

Supplementary Figure 4. Incremental net monetary benefit in the PSM cohort for (A) LYG, (B) QALY

(A)

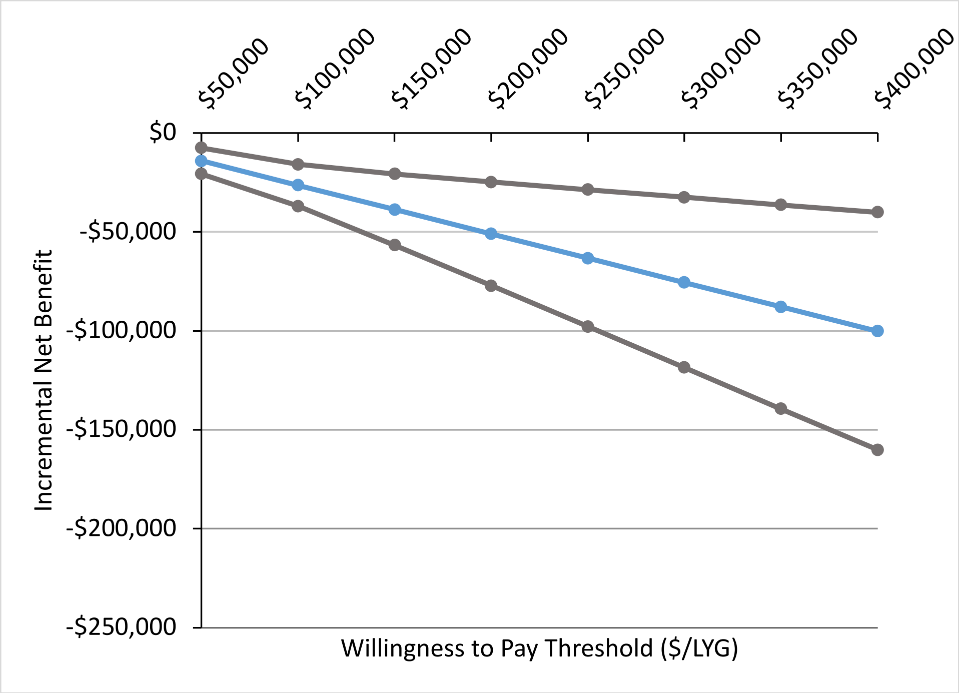

(B)

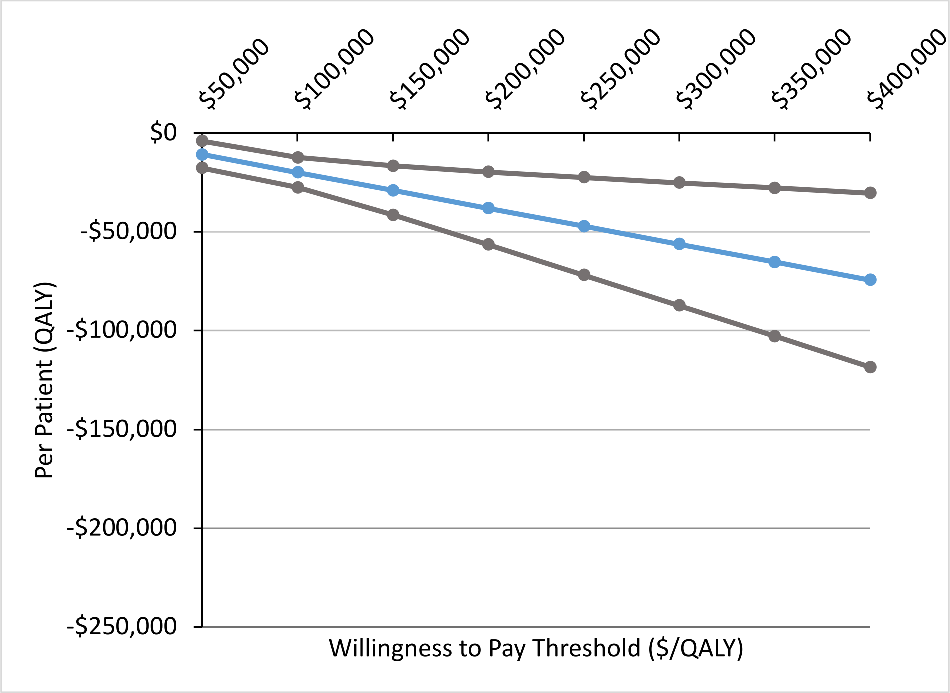

LYG, life-years gained; QALY, quality adjusted life-years; PSM, propensity score matched

CHEERS Checklist
Items to include when reporting economic evaluations of health interventions

The **ISPOR CHEERS Task Force Report**, *Consolidated Health Economic Evaluation Reporting Standards (CHEERS)—Explanation and Elaboration: A Report of the ISPOR Health Economic Evaluations Publication Guidelines Good Reporting Practices Task Force,* provides examples and further discussion of the 24-item CHEERS Checklist and the CHEERS Statement. It may be accessed via the *Value in Health* or via the ISPOR Health Economic Evaluation Publication Guidelines – CHEERS: Good Reporting Practices webpage: http://www.ispor.org/TaskForces/EconomicPubGuidelines.asp

Section/item	Item No	Recommendation	Reported on page No/ line No
**Title and abstract**			
Title	1	Identify the study as an economic evaluation or use more specific terms such as "cost-effectiveness analysis", and describe the interventions compared.	1
Abstract	2	Provide a structured summary of objectives, perspective, setting, methods (including study design and inputs), results (including base case and uncertainty analyses), and conclusions.	3, 4
**Introduction**			
Background and objectives	3	Provide an explicit statement of the broader context for the study.<br>Present the study question and its relevance for health policy or practice decisions.	5-7
**Methods**			
Target population and subgroups	4	Describe characteristics of the base case population and subgroups analysed, including why they were chosen.	7, 8
Setting and location	5	State relevant aspects of the system(s) in which the decision(s) need(s) to be made.	7, 8
Study perspective	6	Describe the perspective of the study and relate this to the costs being evaluated.	7
Comparators	7	Describe the interventions or strategies being compared and state why they were chosen.	7, 8
Time horizon	8	State the time horizon(s) over which costs and consequences are being evaluated and say why appropriate.	7, 8
Discount rate	9	Report the choice of discount rate(s) used for costs and outcomes and say why appropriate.	10
Choice of health outcomes	10	Describe what outcomes were used as the measure(s) of benefit in the evaluation and their relevance for the type of analysis performed.	9-10
Measurement of effectiveness	11a	*Single study-based estimates:* Describe fully the design features of the single effectiveness study and why the single study was a sufficient source of clinical effectiveness data.	9-10

	11b	*Synthesis-based estimates:* Describe fully the methods used for identification of included studies and synthesis of clinical effectiveness data.	NA
Measurement and valuation of preference based outcomes	12	If applicable, describe the population and methods used to elicit preferences for outcomes.	NA
Estimating resources and costs	13a	*Single study-based economic evaluation:* Describe approaches used to estimate resource use associated with the alternative interventions. Describe primary or secondary research methods for valuing each resource item in terms of its unit cost. Describe any adjustments made to approximate to opportunity costs.	8, 10
	13b	*Model-based economic evaluation:* Describe approaches and data sources used to estimate resource use associated with model health states. Describe primary or secondary research methods for valuing each resource item in terms of its unit cost. Describe any adjustments made to approximate to opportunity costs.	NA
Currency, price date, and conversion	14	Report the dates of the estimated resource quantities and unit costs. Describe methods for adjusting estimated unit costs to the year of reported costs if necessary. Describe methods for converting costs into a common currency base and the exchange rate.	10
Choice of model	15	Describe and give reasons for the specific type of decision-analytical model used. Providing a figure to show model structure is strongly recommended.	NA
Assumptions	16	Describe all structural or other assumptions underpinning the decision-analytical model.	NA
Analytical methods	17	Describe all analytical methods supporting the evaluation. This could include methods for dealing with skewed, missing, or censored data; extrapolation methods; methods for pooling data; approaches to validate or make adjustments (such as half cycle corrections) to a model; and methods for handling population heterogeneity and uncertainty.	9
**Results**			
Study parameters	18	Report the values, ranges, references, and, if used, probability distributions for all parameters. Report reasons or sources for distributions used to represent uncertainty where appropriate. Providing a table to show the input values is strongly recommended.	11, Table 1
Incremental costs and outcomes	19	For each intervention, report mean values for the main categories of estimated costs and outcomes of interest, as well as mean differences between the comparator groups. If applicable, report incremental cost-effectiveness ratios.	11-13, Table 2
Characterising uncertainty	20a	*Single study-based economic evaluation:* Describe the effects of sampling uncertainty for the estimated incremental cost and incremental effectiveness parameters, together with the impact	10

		of methodological assumptions (such as discount rate, study perspective).	
	20b	*Model-based economic evaluation:* Describe the effects on the results of uncertainty for all input parameters, and uncertainty related to the structure of the model and assumptions.	NA
Characterising heterogeneity	21	If applicable, report differences in costs, outcomes, or cost-effectiveness that can be explained by variations between subgroups of patients with different baseline characteristics or other observed variability in effects that are not reducible by more information.	NA
**Discussion**			
Study findings, limitations, generalisability, and current knowledge	22	Summarise key study findings and describe how they support the conclusions reached. Discuss limitations and the generalisability of the findings and how the findings fit with current knowledge.	13-15
**Other**			
Source of funding	23	Describe how the study was funded and the role of the funder in the identification, design, conduct, and reporting of the analysis. Describe other non-monetary sources of support.	15
Conflicts of interest	24	Describe any potential for conflict of interest of study contributors in accordance with journal policy. In the absence of a journal policy, we recommend authors comply with International Committee of Medical Journal Editors recommendations.	No authors have conflicts of interest to report

For consistency, the CHEERS Statement checklist format is based on the format of the CONSORT statement checklist

The **ISPOR CHEERS Task Force Report** provides examples and further discussion of the 24-item CHEERS Checklist and the CHEERS Statement. It may be accessed via the *Value in Health* link or via the ISPOR Health Economic Evaluation Publication Guidelines – CHEERS: Good Reporting Practices webpage: http://www.ispor.org/TaskForces/EconomicPubGuidelines.asp

The citation for the CHEERS Task Force Report is:
Husereau D, Drummond M, Petrou S, et al. Consolidated health economic evaluation reporting standards (CHEERS)—Explanation and elaboration: A report of the ISPOR health economic evaluations publication guidelines good reporting practices task force. Value Health 2013;16:231-50.

The RECORD statement for pharmacoepidemiology (RECORD-PE) checklist of items, extended from the STROBE and RECORD statements, which should be reported in non-interventional pharmacoepidemiological studies using routinely collected health data

Item No	STROBE items	RECORD items	RECORD-PE items	Page No
**Title and abstract**				
1	(a) Indicate the study's design with a commonly used term in the title or the abstract.<br>(b) Provide in the abstract an informative and balanced summary of what was done and what was found.	1.1: The type of data used should be specified in the title or abstract. When possible, the name of the databases used should be included.<br>1.2: If applicable, the geographical region and timeframe within which the study took place should be reported in the title or abstract.<br>1.3: If linkage between databases was conducted for the study, this should be clearly stated in the title or abstract.	—	1, 3-4
**Introduction**				
Background rationale				
2	Explain the scientific background and rationale for the investigation being reported.	—	—	5-6
Objectives				
3	State specific objectives, including any prespecified hypotheses.	—	—	6-7
**Methods**				
Study design				
4	Present key elements of study design early in the paper.	—	4.a: Include details of the specific study design (and its features) and report the use of multiple designs if used.<br>4.b: The use of a diagram(s) is recommended to illustrate key aspects of the study design(s), including exposure, washout, lag and observation periods, and covariate definitions as relevant.	7
Setting				
5	Describe the setting, locations, and relevant dates, including periods of recruitment, exposure, follow-up, and data collection.	—	—	7
Participants				
6	(a) Cohort study—give the eligibility criteria, and the sources and methods of selection of participants. Describe methods of follow-up. Case-control study—give the eligibility criteria, and the sources and methods of case ascertainment and control selection. Give the rationale for the choice of cases and controls. Cross sectional study—give the eligibility criteria, and the sources and methods of selection of participants.<br>(b) Cohort study—for matched studies, give matching criteria and number of exposed and unexposed. Case-control study—for matched studies, give matching	6.1: The methods of study population selection (such as codes or algorithms used to identify participants) should be listed in detail. If this is not possible, an explanation should be provided.<br>6.2: Any validation studies of the codes or algorithms used to select the population should be referenced. If validation was conducted for this study and not published elsewhere, detailed methods and results should be provided.<br>6.3: If the study involved linkage of databases, consider use of a flow	6.1.a: Describe the study entry criteria and the order in which these criteria were applied to identify the study population. Specify whether only users with a specific indication were included and whether patients were allowed to enter the study population once or if multiple entries were permitted. See explanatory document for guidance related to matched designs.	7-8

	criteria and the number of controls per case.	diagram or other graphical display to demonstrate the data linkage process, including the number of individuals with linked data at each stage.		
Variables				
7	Clearly define all outcomes, exposures, predictors, potential confounders, and effect modifiers. Give diagnostic criteria, if applicable.	7.1: A complete list of codes and algorithms used to classify exposures, outcomes, confounders, and effect modifiers should be provided. If these cannot be reported, an explanation should be provided.	7.1.a: Describe how the drug exposure definition was developed.<br>7.1.b: Specify the data sources from which drug exposure information for individuals was obtained.<br>7.1.c: Describe the time window(s) during which an individual is considered exposed to the drug(s). The rationale for selecting a particular time window should be provided. The extent of potential left truncation or left censoring should be specified.<br>7.1.d: Justify how events are attributed to current, prior, ever, or cumulative drug exposure.<br>7.1.e: When examining drug dose and risk attribution, describe how current, historical or time on therapy are considered.<br>7.1.f: Use of any comparator groups should be outlined and justified.<br>7.1.g: Outline the approach used to handle individuals with more than one relevant drug exposure during the study period.	8-10
Data sources/measurement				
8	For each variable of interest, give sources of data and details of methods of assessment (measurement). Describe comparability of assessment methods if there is more than one group.	—	8.a: Describe the healthcare system and mechanisms for generating the drug exposure records. Specify the care setting in which the drug(s) of interest was prescribed.	8, 9
Bias				
9	Describe any efforts to address potential sources of bias.	—	—	9
Study size				
10	Explain how the study size was arrived at.	—	—	7
Quantitative variables				
11	Explain how quantitative variables were handled in the analyses. If applicable, describe which groupings were chosen, and why.	—	—	9-10
Statistical methods				
12	(a) Describe all statistical methods, including those used to control for confounding.<br>(b) Describe any methods used to examine subgroups and interactions.<br>(c) Explain how missing data were addressed.<br>(d) Cohort study—if applicable, explain how loss to follow-up was addressed. Case-control study—if applicable, explain how matching of cases and controls was addressed. Cross sectional study—if	—	12.1.a: Describe the methods used to evaluate whether the assumptions have been met.<br>12.1.b: Describe and justify the use of multiple designs, design features, or analytical approaches.	9-11

	applicable, describe analytical methods taking account of sampling strategy.<br>(e) Describe any sensitivity analyses.			
Data access and cleaning methods				
12	—	12.1: Authors should describe the extent to which the investigators had access to the database population used to create the study population.<br>12.2: Authors should provide information on the data cleaning methods used in the study.	—	7
Linkage				
12	—	12.3: State whether the study included person level, institutional level, or other data linkage across two or more databases. The methods of linkage and methods of linkage quality evaluation should be provided.	—	7-8
**Results**				
Participants				
13	(a) Report the numbers of individuals at each stage of the study (eg, numbers potentially eligible, examined for eligibility, confirmed eligible, included in the study, completing follow-up, and analysed).<br>(b) Give reasons for non-participation at each stage.<br>(c) Consider use of a flow diagram.	13.1: Describe in detail the selection of the individuals included in the study (that is, study population selection) including filtering based on data quality, data availability, and linkage. The selection of included individuals can be described in the text or by means of the study flow diagram.	—	11, Figure 1
Descriptive data				
14	(a) Give characteristics of study participants (eg, demographic, clinical, social) and information on exposures and potential confounders.<br>(b) Indicate the number of participants with missing data for each variable of interest.<br>(c) Cohort study—summarise follow-up time (eg, average and total amount).	—	—	11, Table 1
Outcome data				
15	Cohort study—report numbers of outcome events or summary measures over time. Case-control study—report numbers in each exposure category, or summary measures of exposure. Cross sectional study—report numbers of outcome events or summary measures.	—	—	11, 12, Table 2
Main results				
16	(a) Give unadjusted estimates and, if applicable, confounder adjusted estimates and their precision (eg, 95% confidence intervals). Make clear which confounders were adjusted for and why they were included.<br>(b) Report category boundaries when continuous variables are categorised.	—	—	12, Table 2, Figure 2, Table 3

	(c) If relevant, consider translating estimates of relative risk into absolute risk for a meaningful time period.			
Other analyses				
17	Report other analyses done—eg, analyses of subgroups and interactions, and sensitivity analyses.	—	—	12, 13, Appendix
**Discussion**				
Key results				
18	Summarise key results with reference to study objectives.	—	—	13
Limitations				
19	Discuss limitations of the study, taking into account sources of potential bias or imprecision. Discuss both direction and magnitude of any potential bias.	19.1: Discuss the implications of using data that were not created or collected to answer the specific research question(s). Include discussion of misclassification bias, unmeasured confounding, missing data, and changing eligibility over time, as they pertain to the study being reported.	19.1.a: Describe the degree to which the chosen database(s) adequately captures the drug exposure(s) of interest.	14-15
Interpretation				
20	Give a cautious overall interpretation of results considering objectives, limitations, multiplicity of analyses, results from similar studies, and other relevant evidence.	—	20.a: Discuss the potential for confounding by indication, contraindication or disease severity or selection bias (healthy adherer/sick stopper) as alternative explanations for the study findings when relevant. **[A: Original text indicated this item was RECORD (ie, not RECORD-PE)?]**	13-14
Generalisability				
21	Discuss the generalisability (external validity) of the study results.	—	—	15
**Other information**				
Funding				
22	Give the source of funding and the role of the funders for the present study and, if applicable, for the original study on which the present article is based.	—	—	15
Accessibility of protocol, raw data, and programming code				
22	—	22.1: Authors should provide information on how to access any supplemental information such as the study protocol, raw data, or programming code.	—	16

RECORD=reporting of studies conducted using observational routinely collected data; RECORD-PE=RECORD for pharmacoepidemiological research; STROBE=strengthening the reporting of observational studies in epidemiology.

*REFERENCE: Langan SM, Schmidt S, Wing K, Ehrenstein V, Nicholls S, Filion K, Klungel O, Petersen I, Sorensen H, Guttmann A, Harron K, Hemkens L, Moher D, Schneeweiss S, Smeeth L, Sturkenboom M, von Elm E, Wang S, Benchimol EI. The REporting of studies Conducted using Observational Routinely-collected health Data (RECORD) Statement for Pharmacoepidemiology (RECORD-PE). *BMJ* 2018; 363: k3532.

Structured Template and Reporting Tool for Real World Evidence (START RWE)

Contents

Table 1. Administrative Information 2
Table 2. Version History 3
Figure 1. Design Diagram 4
Table 3. Summary of Study Population Parameters 5
A. Meta-data about data source and software 5
B. B. Index date (day 0) defining criterion 7
C. Inclusion criteria 8
D. Exclusion criteria 9
E. Predefined covariates 10
F. Empirically defined covariates 11
G. Outcome 12
H. Follow up 13
Table 4. Primary, Secondary, and Subgroup Analysis Specification 14
A. Primary analysis 14
B. Secondary Analysis 1 14
Table 5. Sensitivity Analyses 15
Table 6. Attrition Table 16
Table 7. Power and Sample Size Calculation 17
Table 8. Glossary of Terminology 18
Table 9. Abbreviations 19

TABLE 1. ADMINISTRATIVE INFORMATION

Instructions: Fill in the yellow highlighted sections where applicable.

Protocol Title:

Real-world cost-effectiveness of first-line gemcitabine + nab-paclitaxel versus FOLFIRINOX in patients with advanced pancreatic cancer: A population-based retrospective cohort in Ontario, Canada

Objective:
Include PICOTS (Patient, Intervention, Comparator, Outcome, Time-Horizon, Setting)

Primary: To evaluate the cost-effectiveness over a 5-year period of initiation gemcitabine + nab-paclitaxel versus FOLFIRINOX (fluorouracil, folinic acid, irinotecan, oxaplatin) for advanced pancreatic cancer

**Protocol registration:**	**Registration identifier**	**Registration date**	**Registration site**
	N/A; study began before START-RWE became available		

**Protocol version:**	**Version number**	**Version date**

Protocol Contributors:

Name	Role	Affiliation
Kelvin Chan	Principal Investigator	Sunnybrook Health Sciences Centre
Jaclyn Beca	Co-Investigator	Ontario Health
Wei Fang Dai	Co-Investigator	University of Toronto
Wanrudee Isaranuwatchai	Co-Investigator	St. Michael's Hospital
Jin Luo	Co-Investigator	ICES
Vanessa Arciero	Co-Investigator	University of Toronto

**Funding:**	**Grant identifier**	**Source**
	N/A	

**Data Use Agreement (DUA)**	**DUA identifier**	**Data provider**	**Data provider contact for data use agreements**
		ICES	

**Human Subjects/Ethics Approval**	**Submission Identifier**	**Date of approval**	**Name of human subjects/ethics approval committee**

TABLE 2. VERSION HISTORY

Instructions: Fill in the yellow highlighted sections to log changes and rationale for making changes made to the prior version of the protocol.

Version date	Version number	Change log	Rationale for change

FIGURE 1. DESIGN DIAGRAM

Instructions: Create design diagram using the framework outlined in Schneeweiss et al. Graphical Depiction of Longitudinal Study Designs in Health Care Databases. Ann Intern Med. 2019;170:398–406. The diagram can be created using power point templates or other software program of choice. It is intended to be read from top to bottom, reflecting the order of operations to create an analytic cohort from a source longitudinal healthcare database. Temporality of assessment windows are clearly shown relative to the cohort entry ("index") date, which is considered day 0. Bracketed number ranges denote the inclusive time windows for washout, inclusion/exclusion, and covariate assessment windows as well as follow up. Whether or not day 0 is included in an assessment window can also be visually distinguished by whether it overlaps the vertical arrow representing the cohort entry date

The diagram may include footnotes specifying the inclusion/exclusion criteria, covariates, and censoring criteria relevant to each assessment window.

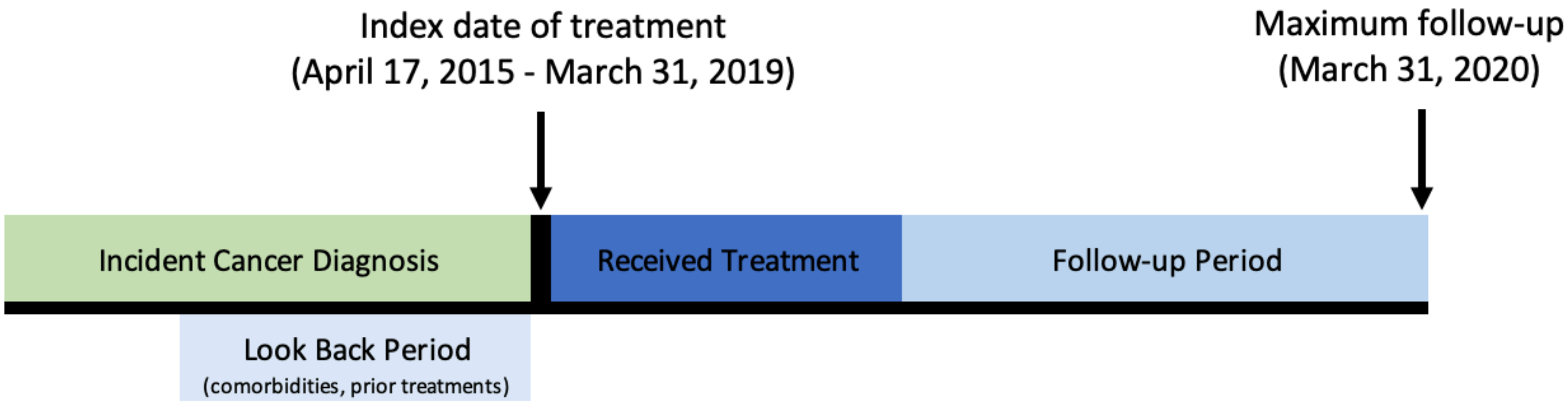

TABLE 3. SUMMARY OF STUDY POPULATION PARAMETERS

Instructions: Fill in the yellow highlighted sections. Example text included.

A. Meta-data about data source and software

This section records the calendar time range used to ascertain cohort entry (index date), as well as the calendar time range of data available for pre-index assessment windows and post-index follow up (study period). The data source name and version are identified, as well as any sampling criteria applied (for example, the data cut only includes patients with a diagnosis of diabetes). If there is data linkage involved, provide a citation or an appendix with description of the linkage (how, performance characteristics)

	**Data Source 1**	**Data Source 2**	**Data Source 3**	**Data Source 4**
**Data source(s):**	New Drug Funding Program	Ontario Cancer Registry	Ontario Health Insurance Plan	Registered Persons Database
**Study period:**	April 17, 2015 – March 31, 2020	April 17, 2015 – March 31, 2020	April 17, 2015 – March 31, 2020	April 17, 2015 – March 31, 2020
**Eligible cohort entry period:**	April 17, 2015 – March 31, 2020	April 17, 2015 – March 31, 2020	April 17, 2015 – March 31, 2020	April 17, 2015 – March 31, 2020
**Data extraction date/version:**				
**Data sampling/extraction criteria:**	All enrollees in data source between April 17, 2015 – March 31, 2020	All enrollees in data source between April 17, 2015 – March 31, 2020	All enrollees in data source between April 17, 2015 – March 31, 2020	All enrollees in data source between April 17, 2015 – March 31, 2020
**Type(s) of data:**	Population-based database	Registry data	Population-based database	Population-based database
**Data linkage:**				
**Data conversion:**				
**Software to create study population:**				

A. Meta-data about data source and software

This section records the calendar time range used to ascertain cohort entry (index date), as well as the calendar time range of data available for pre-index assessment windows and post-index follow up (study period). The data source name and version are identified, as well as any sampling criteria applied (for example, the data cut only includes patients with a diagnosis of diabetes). If there is data linkage involved, provide a citation or an appendix with description of the linkage (how, performance characteristics)

	Data Source 5	Data Source 6	Data Source 7	Data Source 8
**Data source(s):**	Activity Level Reporting Database	Canadian Institutes for Health Information Discharge Abstract Database	Canadian Institutes for Health Information National Ambulatory Reporting System	Canadian Institutes for Health Information Same Day Surgery Database
**Study period:**	April 17, 2015 – March 31, 2020	April 17, 2015 – March 31, 2020	April 17, 2015 – March 31, 2020	April 17, 2015 – March 31, 2020
**Eligible cohort entry period:**	April 17, 2015 – March 31, 2020	April 17, 2015 – March 31, 2020	April 17, 2015 – March 31, 2020	April 17, 2015 – March 31, 2020
**Data extraction date/version:**				
**Data sampling/extraction criteria:**	All enrollees in data source between April 17, 2015 – March 31, 2020	All enrollees in data source between April 17, 2015 – March 31, 2020	All enrollees in data source between April 17, 2015 – March 31, 2020	All enrollees in data source between April 17, 2015 – March 31, 2020
**Type(s) of data:**	Population-based database	Registry data	Population-based database	Population-based database
**Data linkage:**				
**Data conversion:**				
**Software to create study population:**				

Code algorithms for cohort entry date are provided in Appendix A, study entry criteria in Appendix B, covariates in Appendix C and D, outcomes in Appendix E (a * in code algorithm indicates use of a wildcard)

All temporal windows anchored on study population entry date (Day 0) unless otherwise specified.

( ) represent open intervals that do not include the end points

[ ] represent closed intervals that do include the end points

[1] Please enter all that apply. Valid entries: IP = inpatient, OP = outpatient, ED = emergency department, any, other, n/a = not applicable. See Appendix E for details on how care setting is defined

[2] Specify whether a diagnosis code is required to be in the primary position (main reason for encounter)

B. Index Date (day 0) defining criterion

The criterion that define the date of entry to the cohort(s) is specified in this section. There should be one row for each unique definition of study population entry. If the study is descriptive, there may only be one row filled out. An active comparator study may have 2 rows, one for the exposure of interest and one for the comparator.

Check the pre-specified box if the exclusion criterion was specified before beginning data analyses, check the varied for sensitivity box if it was modified as part of sensitivity analyses. Specify the source of algorithms to define study entry criteria.

Study population name(s)	Day 0 Description	Number of entries	Type of entry	Washout window	Care Setting[1]	Code Type	Diagnosis position[2]	Incident with respect to…	Pre-specified	Varied for sensitivity	Source of algorithm
Exposure	Date of incident administration of gemcitabine + nab-paclitaxel for metastatic intent	Single	Incident	N/A	N/A	NDC	N/A	Gemcitabine + nab-paclitaxel (any formulation, metastatic intent)	Yes	No – investigator review of generic names	
Comparator	Date of incident administration of FOLFIRINOX for metastatic intent	Single	Incident	N/A	N/A	NDC	N/A	FOLFIRINOX (any formulation, metastatic intent)	Yes	No – investigator review of generic names	

C. Inclusion Criteria

Describe what the criterion is conceptually. Specify the order of application of the inclusion criteria is relative to selection of the index date (day 0) for study entry. For example, specify “after selection of index date” if you plan to 1) select the index date based on first time the study entry defining criterion is met in the study period, 2) then apply inclusion-exclusion criteria, 3) keep the selected index date for study entry if all inclusion-exclusion criteria are met. Alternatively, you can specify “before selection of index date” if you plan to 1) identify all potential index dates meeting the study entry criterion, 2) apply inclusion-exclusion criteria, 3) select one or more of the study entry dates that meet all inclusion-exclusion criteria. Define the assessment window relative to the index date, whether there are restrictions on care setting or diagnosis position in the algorithm to define each inclusion criterion and specify which study populations (defined in Table 3B) the criterion is applied to.

Defining “observable” patient time in the healthcare data source is almost always required as an inclusion criterion. When using administrative claims data, this can be measured with dates of enrollment in insurance coverage, with or without bridging of short gaps in enrollment. When using electronic health record data, defining observable patient time may require making some strong assumptions. For example, assuming that patient encounters are always observable, that patients are observable between the first and last recorded encounter in the record, that patients are observable for X days before and after any recorded encounter, etc. Alternatively, one could specify inclusion based on algorithms to measure “loyalty” to a healthcare provider or EHR system.

Check the pre-specified box if the exclusion criterion was specified before beginning data analyses, check the varied for sensitivity box if it was modified as part of sensitivity analyses. Specify the source of algorithms to define inclusion criteria.

Criterion	Details	Order of application	Assessment window	Care Settings[1]	Code Type	Diagnosis position[2]	Applied to study populations:	Pre-specified	Varied for sensitivity	Source for algorithm
Observable time	Advanced pancreatic cancer diagnosis	Before selection of index date	[undefined, 0]	N/A	N/A	N/A	Exposure, comparator	Yes	No	N/A
Received study drug of interest		Before selection of index date	[0, 0]	N/A	N/A	N/A	Exposure, comparator	Yes	No	N/A
			...	...			...			...

D. Exclusion Criteria<br>Describe what the criterion is conceptually. Specify the order of application of the exclusion criteria is relative to selection of the index date (day 0) for study entry. Define the assessment window relative to the index date, whether there are restrictions on care setting or diagnosis position in the algorithm to define each exclusion criterion and specify which study populations (defined in Table 3B) the criterion is applied to.<br><br>Check the pre-specified box if the exclusion criterion was specified before beginning data analyses, check the varied for sensitivity box if it was modified as part of sensitivity analyses. Specify the source of algorithms to define exclusion criteria.										
**Criterion**	**Details**	**Order of application**	**Assessment window**	**Care Settings[1]**	**Code Type**	**Diagnosis position[2]**	**Applied to study populations:**	**Pre-specified**	**Varied for sensitivity**	**Source for algorithm**
Age <18 or >105	(Cohort entry date – date of birth)/365	Before selection of index date	[0, 0]	N/A	N/A	N/A	Exposure, comparator	Yes	No	N/A
Received treatment ≥60 days prior to the date of cancer diagnosis		Before selection of index date	[undefined , 0]	N/A	N/A	N/A	Exposure, comparator	Yes	No	N/A
Death prior to index date		After selection of index date	[undefined , 0]	N/A	N/A	N/A	Exposure, comparator	Yes	No	N/A
Non-Ontario resident at the time of diagnosis		Before selection of index date	[undefined , 0]	N/A	N/A	N/A	Exposure, comparator	Yes	No	N/A
Missing income quintile		Before selection of index date	[undefined , 0]	N/A	N/A	N/A	Exposure, comparator	Yes	No	N/A
Missing rurality status		Before selection of index date	[undefined , 0]	N/A	N/A	N/A	Exposure, comparator	Yes	No	N/A
Missing extent of disease (locally advanced versus metastatic)		Before selection of index date	[undefined , 0]	N/A	N/A	N/A	Exposure, comparator	Yes	No	N/A
Eastern Cooperative Oncology Group performance status ≥2 or missing		Before selection of index date	[undefined , 0]	N/A	N/A	N/A	Exposure, comparator	Yes	No	N/A

E. Predefined Covariates

Define the covariate conceptually, with accompanying details as necessary. Specify which planned analyses adjust for the covariate, and how it is specified in the analysis (e.g. continuous, categorical, binary). Define the assessment window relative to the index date (day 0), whether there are restrictions on care setting or diagnosis position in the algorithm, and which study populations defined in Table 3B the covariate is measured for. Specify the source of algorithms to define covariates.

Check the pre-specified box if the covariate was specified before beginning data analyses, check the varied for sensitivity box if it was modified as part of sensitivity analyses. Specify the source of algorithms to define covariates.

Characteristic	Details	Type of variable	Assessment window	Care Settings[1]	Code Type	Diagnosis position[2]	Applied to study populations:	Pre-specified	Varied for sensitivity	Source for algorithm
Age at index	(Index date – date of birth)	Continuous	[0, 0]	N/A	N/A	N/A	Exposure, comparator	Yes	No	N/A
Sex	Male, female, unknown	Categorical	[0, 0]	N/A	N/A	N/A	Exposure, comparator	Yes	No	N/A
Prior cancer diagnosis	Yes, no	Binary	[0, 0]	N/A	N/A	N/A	Exposure, comparator	Yes	No	N/A
Location of pancreatic cancer	Body, head, tail, other	Categorical	[0, 0]	N/A	N/A	N/A	Exposure, comparator	Yes	No	N/A
Extent of disease	Locally advanced, metastatic	Binary	[0, 0]	N/A	N/A	N/A	Exposure, comparator	Yes	No	N/A
Prior pancreatic radiation	Yes, no	Binary	[undefined, 0]	N/A	N/A	N/A	Exposure, comparator	Yes	No	N/A
Adjuvant chemotherapy	Yes, no	Binary	[undefined, 0]	N/A	N/A	N/A	Exposure, comparator	Yes	No	N/A
Prior pancreatic surgery	Yes, no	Binary	[undefined, 0]	N/A	N/A	N/A	Exposure, comparator	Yes	No	N/A
Health region (LHIN)	Region 1 to 14	Categorical	[0, 0]	N/A	N/A	N/A	Exposure, comparator	Yes	No	N/A
Income quintile	Q1 to Q5	Categorical	[0, 0]	N/A	N/A	N/A	Exposure, comparator	Yes	No	N/A
Rurality	Urban, rural	Binary	[0, 0]	N/A	N/A	N/A	Exposure, comparator	Yes	No	N/A
ECOG PS at index	0, 1	Categorical	[0, 0]	N/A	N/A	N/A	Exposure, comparator	Yes	No	N/A
Charlson-Deyo comorbidity index	0, 1, 2+	Categorical	[-1095, 0]	Hospitalization	ICD-10	N/A	Exposure, comparator	Yes	No	N/A
Adjusted Clinical Groups score	0 to 15+	Categorical	[-1095, 0]			N/A	Exposure, comparator	Yes	No	N/A
Time between diagnosis to index date	(Index date – diagnosis date)	Continuous	[undefined, 0]	N/A	N/A	N/A	Exposure, comparator	Yes	No	N/A

Code algorithms for cohort entry date are provided in Appendix A, study entry criteria in Appendix B, covariates in Appendix C and D, outcomes in Appendix E (a * in code algorithm indicates use of a wildcard)
All temporal windows anchored on study population entry date (Day 0) unless otherwise specified.
( ) represent open intervals that do not include the end points
[ ] represent closed intervals that do include the end points
[1] Please enter all that apply. Valid entries: IP = inpatient, OP = outpatient, ED = emergency department, any, other, n/a = not applicable. See Appendix E for details on how care setting is defined
[2] Specify whether a diagnosis code is required to be in the primary position (main reason for encounter)

F. Empirically Defined Covariates									
Empirical identification of covariates to use in confounding control may not be relevant to all study populations or analyses, however if such methods are used, this section includes fields to describe what the algorithm for covariate identification is, as well as specification of the settings or parameters used to empirically identify covariates. In this section, specify the assessment window relative to the index date (day 0), which analyses adjust for empirically identified covariates, how the covariates are specified in a model, whether there are restrictions on care setting or diagnosis position, and which study populations (defined in section 3B) to measure the empirical covariates.<br><br>Check the pre-specified box if the empirical covariate selection parameters were specified before beginning data analyses, check the varied for sensitivity box if the parameters were modified as part of sensitivity analyses. Specify the source for the method and/or software used for empirically defined covariates.									
**Algorithm**	**Type of variable**	**Assessment window**	**Care Settings[1]**	**Code Type**	**Diagnosis position[2]**	**Applied to study populations:**	**Pre-specified**	**Varied for sensitivity**	**Source/code for algorithm**

G. Outcome

Define the outcome conceptually and whether it is the primary outcome of interest. Specify whether the type of outcome is incident (if so, there is a field to specify the washout window to define "incident" occurrences), prevalent or other. Specify whether there are restrictions on care setting or diagnosis position, and which groups or analyses the outcome is measured for. If there are measurement characteristics for the outcome algorithm (e.g. PPV, sensitivity, specificity) from publications, or from outcome validation within the study population (e.g., medical record review), provide this information.

Check the pre-specified box if the outcome parameters were specified before beginning data analyses, check the varied for sensitivity box if the parameters were modified as part of sensitivity analyses. Specify the source of algorithms to define outcomes.

Outcome name	Outcome measurement characteristics	Primary outcome?	Type of outcome	Washout window	Care Settings[1]	Code Category	Diagnosis position[2]	Applied to study populations:	Pre-specified	Varied for sensitivity	Source of algorithm
Incremental cost-effectiveness ratio	Cost and life-years gain or quality-adjusted life-years	Yes	Ratio	N/A	N/A	N/A	Primary	Exposure, comparator	Yes	Yes	
Incremental net monetary benefit	Willingness to pay threshold, cost, life-years gained	No	Continuous	N/A	N/A	N/A	Primary	Exposure, comparator	Yes	Yes	

H. Follow up
Specify when follow up begins relative to the index date (day 0) and select each criterion that is used to end follow up.

Check the pre-specified box if the outcome parameters were specified before beginning data analyses, check the varied for sensitivity box if the parameters were modified as part of sensitivity analyses.

**Begins**	Day 1			
**Ends**	**Select all that apply**	**Specify**	**Pre-specified**	**Varied for sensitivity**
**Date of Outcome**	No	N/A	N/A	N/A
**Date of Death**	Yes		Yes	No
**Date of Disenrollment**	Yes		Yes	No
**Day X following index date (specify date)**	Yes	Day 1825 (5 years maximum)	Yes	No
**End of study period (specify date)**	Yes	March 31, 2020	Yes	No
**End of exposure (specify operational details, e.g. stockpiling, algorithm, grace period)**	No	N/A	N/A	N/A
**Date of add to/switch from exposure (specify algorithm)**	No	N/A	N/A	N/A
**Other (specify)**	No	N/A	N/A	N/A

Code algorithms for cohort entry date are provided in Appendix A, study entry criteria in Appendix B, covariates in Appendix C and D, outcomes in Appendix E (a * in code algorithm indicates use of a wildcard)
All temporal windows anchored on study population entry date (Day 0) unless otherwise specified.
( ) represent open intervals that do not include the end points
[ ] represent closed intervals that do include the end points
¹ Please enter all that apply. Valid entries: IP = inpatient, OP = outpatient, ED = emergency department, any, other, n/a = not applicable. See Appendix E for details on how care setting is defined

TABLE 4. ANALYSIS SPECIFICATIONS
Instructions: Fill in the yellow highlighted sections.

	Primary	Secondary Analysis 1
**Hypothesis:**	Exposure increases cost and lowers benefit relative to comparator	Exposure increases cost and lowers benefit relative to comparator
**Study population(s)**	Exposure, comparator	Exposure, comparator
**Outcome:**	Incremental cost effectiveness ratio	Incremental net monetary benefit
**Software:**	SAS 9.4: Proc Phreg, Proc Means, %getcost Macro at ICES	SAS 9.4: Proc Phreg, Proc Means, %getcost Macro at ICES
**Model(s):**	Outcome model: Ratio<br>(incremental cost between cases vs controls) / (incremental benefit between cases vs controls)<br>Benefit is defined as both life-years gained and quality-adjusted life-years gained<br><br>Propensity score model: Logistic regression<br><br>Exposure =<br><br>Age at index +<br>Sex +<br>Prior cancer diagnosis +<br>Charlson-Deyo comorbidity index +<br>Total ACG score +<br>Health region (LHIN) +<br>Income quintile +<br>Rurality +<br>Time from pancreatic cancer diagnosis +<br>Prior pancreatic surgery +<br>Prior pancreatic radiation +<br>Adjuvant chemotherapy +<br>Extent of disease +<br>ECOG PS +<br>Index year	Outcome model: Continuous<br>The net benefit (NB), $NB = (effect_i \times \lambda) - cost_i$, for each person (i) was calculated for the willingness to pay threshold (λ) beginning at $50,000<br><br>Propensity score model: Logistic regression<br><br>Exposure =<br><br>Age at index +<br>Sex +<br>Prior cancer diagnosis +<br>Charlson-Deyo comorbidity index +<br>Total ACG score +<br>Health region (LHIN) +<br>Income quintile +<br>Rurality +<br>Time from pancreatic cancer diagnosis +<br>Prior pancreatic surgery +<br>Prior pancreatic radiation +<br>Adjuvant chemotherapy +<br>Extent of disease +<br>ECOG PS +<br>Index year
**Confounding adjustment method:**	Propensity score matching – nearest neighbor, ratio = fixed 1:1, caliper 0.02<br>Propensity score weighting	Propensity score matching – nearest neighbor, ratio = fixed 1:1, caliper 0.02<br>Propensity score weighting
**Missing data methods:**	Missing indicators	Assumption that if no relevant claims diagnoses/procedures are present, the condition is not present

TABLE 5 SENSITIVITY ANALYSES
Instructions: Fill in the yellow highlighted sections.

	What is the parameter being varied? (be clear what it is changing from)	Why? (What do you expect to learn?)	Strengths of the sensitivity analysis compared to the primary?	Weaknesses of the sensitivity analysis compared to the primary?
**Sensitivity Analysis 1**<br>**Sensitivity Analysis 2**<br>**Sensitivity Analysis 3**	Propensity score matched (PSM) cohort	To test for robustness of the primary analysis (IPTW cohort)	An additional method of balancing experimental and control arms, patients matched 1:1	

TABLE 5. ATTRITION TABLE

Instructions: Fill in the yellow highlighted sections. Show the number of patients remaining after applying each inclusion/exclusion criterion, sequentially.

	**Total Cohort**		**Drug A**		**Drug B**	
	**Excluded Patients**	**Remaining Patients**	**Excluded Patients**	**Remaining Patients**	**Excluded Patients**	**Remaining Patients**
All patients with advanced pancreatic cancer who began treatment with gemcitabine + nab-paclitaxel, FOLFIRINOX, or gemcitabine after April 17, 2015	N/A	2732				
Age <18 years	0	2732				
Non-Ontario resident	0	2732				
Death prior to index date	0	2732				
No pancreatic cancer diagnosis within 60 days of treatment initiation	234	2498				
Received gemcitabine alone	300	2198				
Missing income quintile	210	1988				
Missing rurality						
Missing disease status or ECOG PS						
ECOG PS ≥2						
			Drug A initiators N=928		Drug B initiators N=1060	

TABLE 7. POWER AND SAMPLE SIZE CALCULATION

Instructions: Fill in the yellow highlighted sections.
Specify the software used, what is being calculated (e.g. power, sample size, detectable difference), the population and statistical assumptions for the calculations. For each parameter assumption, specify the primary assumption and the range considered. Specify the sources used to select the estimated population parameters. The power or sample size calculations across the range of assumed parameter values may be displayed in tabular or visual form as needed.

The template contains assumptions to calculate power for a comparison of 2 proportions, however the entries for the population assumptions and statistical parameters should be modified to reflect those that are relevant for the calculation used by the investigators.

**Software:**	powersamplesize.com
**Calculate:**	Power to compare 2 proportions: 2-Sample, 2-Sided Equality

**Population assumptions**	**Primary**	**Range**	**Source for estimated parameters**
**Statistical parameters:**			

TABLE 8 GLOSSARY

Term	Definition
Confounder	Variable other than the exposure of interest or outcome under investigation that is 1) associated with exposure, 2) a risk factor for the outcome, and 3) not on the causal pathway between the exposure and the outcome. A confounder can artificially inflate or reduce the magnitude of association between an exposure and outcome.
Covariates	Variables that are neither exposure nor outcome of interest, but are measured to describe a population or because they may be a confounder to account for in analyses
Data Extraction Date	The date when the data were extracted from the dynamic healthcare database
Days Supplied	Number of days supplied for a dispensed prescription
Eligible cohort entry period	Calendar time frame during which cohort entry dates are identified
Empirically defined covariates	Covariates that are not prespecified by the investigator. The selection of these covariates is based on applying algorithms to the data. The algorithms for covariate selection may be tuned by investigator specified parameters.
Observable Time	For insurance claims data, this may refer to periods of enrollment in medical and/or drug plans. For electronic health record databases, this may refer to algorithms designed to identify patients whose healthcare contacts are likely to be covered within the healthcare system.
Observable Time Gap	Maximum number of days allowed between two consecutive observable time windows to still be considered “continuously observable”.
Follow up window	The interval during which occurrence of the outcome of interest in the study population will be included in the analysis.
Grace Window	Number of days added to days supply to allow for non-adherence or account for the hypothesized biologic exposure risk window. Operationally, this could be defined as the number of extra days added to the end of a days supply to extend time counted as "exposed". This grace may bridge the gap between dispensations where the days supply dispensed does not cover all days until the refill.
Index Date	The date when subjects enter the study population (cohort entry date, outcome event date). It is defined based on events in the patient's longitudinal timeline, other windows are defined relative to the index date.
Assessment Window	Interval during which a patient is required to have evidence of a pre-existing condition (diagnosis/procedure/drug dispensing). May be used for washout of exposure or outcome, exclusion assessment or covariate assessment.
Predefined Covariates	Covariates that are prespecified and defined by the investigator in the protocol.
Principal Diagnosis	Diagnosis or condition established to be chiefly responsible for admission of the patient to the hospital.
Source Data Range	The calendar time range covered by a data source that is available
Stockpiling Algorithm	Algorithm defining how early refills are handled when determining length of exposure follow up
Study Period	Calendar time interval of data available for study, including pre-index date assessment windows and post-index follow up
Treatment Episode	Continuous period of exposure defined using by dispensation date + days supply and applying stockpiling algorithms and/or grace windows
Washout Window	Minimum number of days a patient is required to have no evidence of prior exposure and/or outcome
Wildcard	Symbol used to represent any single alphanumeric digit in code algorithms. For example 410.*1, where * is the wildcard.

ABBREVIATIONS

RX = drug prescription/dispensation
DX = diagnosis
PX = procedure
LB = laboratory
NDC = national drug code
ICD-9-CM = International Classification of Diseases - Clinical Modification 9th revision (2012)
ICD-10-CM = ICD-CM 10th revision
ICD-11-CM = ICD-CM 11th revision
CPT = Current Procedural Terminology
HCPCS = Healthcare Common Procedure Coding System
LOINC = Logical Observation Identifiers Names and Codes
IP = inpatient
OP = outpatient
ED = emergency department
N/A = not applicable
